# Mortality, Criminal Sanctions, and Court Diversion in People With Psychosis

**DOI:** 10.1001/jamanetworkopen.2024.42146

**Published:** 2024-10-31

**Authors:** Erin Spike, Preeyaporn Srasuebkul, Tony Butler, Julian Trollor, Jocelyn Jones, Kyllie Cripps, Grant Sara, Luke Grant, Stephen Allnutt, David Greenberg, Peter W. Schofield, Armita Adily, Nabila Zohora Chowdhury, Azar Kariminia

**Affiliations:** 1 The Kirby Institute, Faculty of Medicine and Health, University of New South Wales Sydney, Sydney, New South Wales, Australia; 2 The National Centre for Excellence in Intellectual Disability Health, Faculty of Medicine and Health, University of New South Wales Sydney, Sydney, New South Wales, Australia; 3 School of Population Health, Faculty of Medicine and Health, University of New South Wales Sydney, Sydney, New South Wales, Australia; 4 National Drug Research Institute, Curtin University, Perth, Western Australia, Australia; 5 Monash Indigenous Studies Centre, Monash University, Melbourne, Victoria, Australia; 6 School of Law, Society and Criminology, University of New South Wales Sydney, Sydney, New South Wales, Australia; 7 School of Psychiatry, Faculty of Medicine and Health, University of New South Wales Sydney, Sydney, New South Wales, Australia; 8 InforMH, Ministry of Health, Sydney, New South Wales, Australia; 9 Corrective Services New South Wales, Sydney, New South Wales, Australia; 10 Justice Health & Forensic Mental Health Network, New South Wales Health, Sydney, New South Wales, Australia; 11 Department of Neuropsychiatry, Hunter New England Local Health District, Newcastle, New South Wales, Australia; 12 Hunter Medical Research Institute, University of Newcastle, Newcastle, New South Wales, Australia

## Abstract

**Importance:**

People living with psychosis experience excess premature mortality and are overrepresented in criminal legal systems, but little is known about mortality associated with criminal sanctions or diversion in this population.

**Objective:**

To examine associations of different types of recent (past 2 years) criminal sanction, including court diversion, with mortality among people with psychosis.

**Design, Setting, and Participants:**

This population-based, retrospective, data-linkage cohort study was conducted using 6 routinely collected administrative data collections from New South Wales, Australia, relating to health, court proceedings, imprisonment, and mortality. Participants (adults aged ≥18 years hospitalized for psychotic disorders) entered observation at the time of discharge from their first psychosis-related hospital admission (or their 18th birthday if aged <18 years) between July 2001 and November 2017 and were followed-up until May 2019. Data were analyzed between February 2023 and April 2024.

**Exposures:**

Recent (past 2 years) criminal sanction type, a time-varying variable with 5 categories: no recent criminal sanction, recent mental health court diversion, recent community sanction, current imprisonment, and recent prior imprisonment (ie, recent prison release).

**Main Outcomes and Measures:**

Causes of death were described, and age- and sex-specific mortality rates by recent criminal sanction type were calculated. In those younger than 65 years, Cox regression was used to examine associations of all-cause and external-cause mortality with recent criminal sanction type, adjusting for sociodemographic, health-related, and offense-related confounders.

**Results:**

The cohort included 83 071 persons (35 791 female [43.1%]; 21 208 aged 25-34 years [25.5%]; median [IQR] follow-up, 9.5 [4.8-14.2] years), of whom 25 824 (31.1%) received a criminal sanction. There were 11 355 deaths. In those aged younger than 65 years, recent mental health court diversion, community sanctions, and prior imprisonment were associated with increased hazards of all-cause and external-cause mortality compared with no recent sanction, with the largest adjusted hazard ratios (aHRs) observed for recent prior imprisonment (all-cause mortality: aHR, 1.69; 95% CI, 1.50-1.91; external-cause mortality: aHR, 2.64; 95% CI, 2.27-3.06).

**Conclusions and Relevance:**

In this cohort study of people with psychosis, recent criminal sanctions were associated with increased mortality irrespective of sanction type. These findings suggest that future research should develop strategies to improve health and safety in people with psychosis who have criminal legal system contact.

## Key Points

**Question:**

Are recent criminal sanctions or court diversion associated with mortality in people with psychosis?

**Findings:**

In this cohort study of 83 071 adults with psychosis, compared with no recent criminal sanction, recent mental health court diversion, community sanctions, and prior imprisonment were associated with a statistically significant increase in the hazards of all-cause and external-cause mortality.

**Meaning:**

These findings suggest that mortality is elevated among people with psychosis following receipt of criminal sanctions, indicating a need for effective interventions and models of care to improve the health and safety of this group.

## Teaser

This cohort study analyzes associations of criminal sanctions with mortality among people with psychosis in the criminal legal system of New South Wales, Australia.

## Introduction

People living with psychosis experience greatly increased premature mortality compared with their peers.^1,2^ Criminal legal system (CLS) involvement may be one important risk factor, given the overrepresentation of people with psychosis in prisons^3^ and CLSs worldwide.^4^ In general population studies, imprisonment^5^ and community sanctions^6^ are associated with increased age- and sex-adjusted mortality risks, with particularly elevated risks of fatal overdose and suicide after prison release.^5,7^ Similarly, North American^8^ and European^9,10,11^ studies among cohorts with psychosis or serious mental illness (SMI) report increased risks of all-cause mortality^9,12^ and suicide^8,10,11^ associated with either criminal convictions^8,9,10,11^ or imprisonment,^12^ although a Danish study^13^ found no association of criminal sentencing to psychiatric treatment with all-cause mortality.

Globally, many jurisdictions provide alternative CLS pathways for people with SMI, including at trial (eg, fitness considerations and diminished or nonresponsibility findings) and/or disposition (eg, secure placement or sentencing to psychiatric treatment).^14,15,16^ Numerous jurisdictions, including in North America, the UK, and Australia, have also introduced mechanisms such as court diversion programs and mental health courts to divert people with SMI accused of less serious offenses into alternative pathways, often including psychiatric treatment.^17,18^ Aims include reducing recidivism and improving health and treatment access.^18^

With previous studies examining single sanction types^12,13^ or criminal convictions,^8,9,10,11^ it is unclear whether mortality risks in people with psychosis vary by sanction type, including diversionary alternatives. To address this, we aimed to investigate associations of different types of recent (past 2 years) criminal sanctions—including mental health court diversion—with mortality among people with psychosis in New South Wales (NSW), Australia between 2001 and 2019. We focused on a 2-year time frame because mortality risks associated with CLS involvement decline beyond this time point,^5^ with later deaths likely reflecting extraneous factors. Specifically, we aimed to (1) describe rates and causes of mortality by recent criminal sanction type and (2) examine associations of recent criminal sanction type with all-cause and external-cause mortality among people with psychosis.

## Methods

### Study Design

This population-based, retrospective, data-linkage cohort study was approved with a waiver of informed consent by the NSW Population and Health Services Research Ethics Committee, and the ethics committees of the Aboriginal Health and Medical Research Council, Corrective Services NSW (CSNSW), and the NSW Justice Health and Forensic Mental Health Network. The reporting of the study followed the Strengthening the Reporting of Observational Studies in Epidemiology (STROBE) reporting guideline.^19^ We used 6 routinely collected administrative data collections from NSW, Australia (July 2001 to May 2019 unless stated): the Admitted Patient Data Collection (APDC), recording public and private hospital admissions; the Emergency Department Data Collection (EDDC), recording ED presentations (January 2005 to May 2019); the NSW Bureau of Crime Statistics and Research Reoffending Database (RoD), recording criminal charges, convictions, and penalties (minor traffic offenses excluded); the CSNSW Offender Integrated Management System (OIMS), recording imprisonments in adult prisons; and the NSW Register of Births, Deaths, and Marriages death registrations (RBDM-DR) and Australian Coordinating Registry Cause of Death Unit Record File (COD-URF), recording deaths and (COD-URF only) causes of deaths registered in NSW.

### Study Population

**Figure 1.**
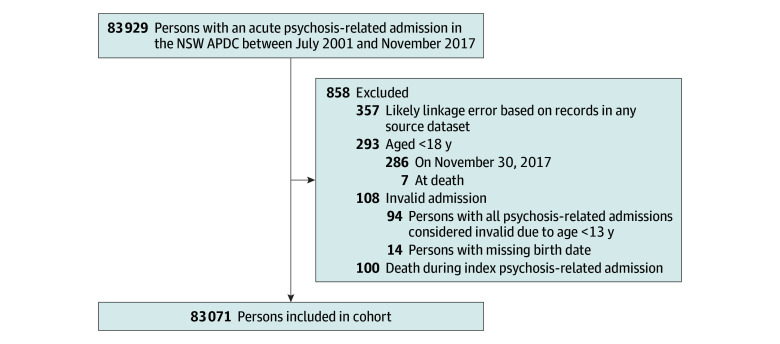
Participant Selection Flow Diagram APDC indicates Admitted Patient Data Collection; NSW, New South Wales.

Our cohort included all adults (≥18 years) discharged from an acute psychosis-related hospital admission recorded in the APDC from July 1, 2001, to November 30, 2017. Participants entered the study at discharge from their first (index) psychosis-related admission during this period or their 18th birthday if younger than 18 years at discharge (provided this was before November 30, 2017). We analyzed follow-up data to May 31, 2019. Psychosis-related admissions were those with a principal diagnosis (*International Statistical Classification of Diseases and Related Health Problems, Tenth Revision [ICD-10]*) of organic and puerperal psychoses (F06.0, F06.2, or F53.1), substance-induced psychoses (F1x.5 [x represents a wildcard character]), schizophrenia spectrum disorders (F20, or F22-F29), and/or affective disorders with psychotic symptoms (F30.2, F31.2, F31.5, F32.3, or F33.3). We excluded admissions at younger than 13 years due to potential diagnostic uncertainty. We excluded participants with suspected linkage error (see eAppendix 1 in Supplement 1) or who died during their index admission (Figure 1).

### Procedures

The NSW Centre for Health Record Linkage performed the data linkage using ChoiceMaker software version 2.7.2 (ChoiceMaker, LLC), employing a probabilistic method with a targeted 0.5% false-positive rate.^20^ We received only deidentified data. Information on exclusion criteria for individual records is available in eAppendix 2 in Supplement 1.

### Outcomes

The primary outcome was all-cause mortality (RBDM-DR and COD-URF). The secondary outcome was external-cause mortality (COD-URF; ie, deaths with an underlying cause of psychoactive substance use [*ICD-10* codes F11-F19], abuse of nondependence-producing substances [*ICD-10* code F55], or external causes of morbidity and mortality [*ICD-10* Chapter XX]). We also identified deaths due to accidental drug overdose (*ICD-10* codes F11-F16, F18, F19, or X40-X44), suicide including undetermined intent (*ICD-10* codes X60-X84, Y10-Y34, Y87.0, or Y87.2), undetermined or unknown cause (*ICD-10* code R99 or missing) and disease-related causes (deaths not categorized as external-cause or undetermined or unknown).

### Main Exposure

The main exposure was recent (past 2 years) adult criminal sanction type, a time-varying variable with 5 mutually exclusive categories: no recent criminal sanction, recent mental health court diversion, recent community sanction, current imprisonment, and recent prior imprisonment (ie, recent prison release). Participants’ exposure category on any given follow-up date reflected sanctions received in the preceding 2 years. To account for multiple simultaneous sanctions and known differences in mortality risks in prison vs community settings,^7,21,22^ we applied a hierarchical classification whereby current imprisonment overrode all other categories; prior imprisonment and mental health court diversion overrode community sanctions, and mental health court diversion and prior imprisonment overrode each other depending on which was more recent.

We ascertained adult community sanctions (court-ordered penalties such as fines, community service, and supervision orders) and mental health court diversion (charge dismissal under Sections 32 or 33 of the NSW *Mental Health [Forensic Provisions] Act 1990*^23^) from the RoD. Sections 32 and 33 enabled the lower courts to dismiss relatively minor (summary) charges against defendants with mental illness and order that they receive assessment, treatment, or support (see eAppendix 3 in Supplement 1). We excluded juvenile-specific sanctions and sanctions or diversions finalized in a juvenile jurisdiction or before the 18th birthday. We ascertained current and prior adult imprisonment (any imprisonment, remand or sentenced, and in an adult prison on or after the 18th birthday, including those commencing before and continuing on this date) from OIMS; prior imprisonment commenced the day after each imprisonment end date. We defined no recent criminal sanction as no community sanction, mental health court diversion, or imprisonment in the preceding 2 years. A small number of participants (616 participants) received not guilty by reason of mental illness verdicts or other or unspecified mental health dismissals; we did not consider these criminal sanctions, except local court dismissals before 2005, which were not differentiated from Section 32 or 33 dismissals. See eAppendix 4 in Supplement 1 for further detail regarding exposure.

### Covariates

We ascertained sex from the APDC, EDDC, RoD, and COD-URF. We ascertained Aboriginal and/or Torres Strait Islander (hereafter, respectfully, Aboriginal) identity from the APDC, EDDC, RoD, OIMS, and COD-URF using a validated multistage median algorithm.^24^ We calculated time-updated age using birthdates in the APDC, EDDC, RoD, and RBDM-DR, grouped into year bands (18-24, 25-34, 35-44, 45-54, 55-64, and ≥65 years). We ascertained marital status, residential Index of Relative Socioeconomic Disadvantage (IRSD), and residential remoteness at index admission from the APDC, mapping 2011 Statistical Local Area codes to the Australian Bureau of Statistics 2011 IRSD scores^25^ and Australian Statistical Geography Standard^26^ to derive IRSD (reclassified into tertiles within NSW) and remoteness, respectively. We ascertained history of problematic drug use (*ICD-10* codes F11-F16, F18-F19, T40, or T43.6; *International Classification of Diseases, Ninth Revision [ICD-9] *codes 292, 304, or 305.2-305.9) and alcohol use (*ICD-10* codes F10, T51, X45, X65, Y15, or Y90-91; or *ICD-9* codes 291, 303, or 305.0) at study entry from the APDC and EDDC. Some EDDC diagnoses used Systematized Nomenclature for Medicine–Clinical Terminology (Australian release) codes, which we selected using the US National Library of Medicine Unified Medical Language System map^27^ and PyMedTermino2 software^28^ with Python version 3.9 (Python Software Foundation) followed by manual review (see eAppendix 5 in Supplement 1). We ascertained past-year Charlson Comorbidity Index (CCI),^29^ a validated physical comorbidity score from 0 to 24, from the APDC (reclassified into 0, 1-2, and ≥2) as a time-varying covariate updated every birthday. We ascertained whether the index admission was voluntary or involuntary (a proxy for psychosis severity) from the APDC. We ascertained offense history at study entry from the RoD using juvenile and adult offenses that received a guilty or not guilty by reason of mental illness verdict or mental health dismissal, categorized as violent (Australian and New Zealand Society of Criminology offense groups 1-6^30^), nonviolent only (offense groups 7-16), and none. Covariates were identified as potential confounders a priori, informed by a directed acyclic graph (see eAppendix 6 in Supplement 1).^31^ Unknown or missing values were minimal (<3% for all covariates except marital status) and were combined with the most common category.

### Statistical Analysis

We calculated age- and sex-specific mortality rates per 1000 person-years with 95% CIs using a Poisson distribution and proportions of deaths by cause, descriptively. For both sexes separately and combined, we modeled associations of recent criminal sanction type with all-cause and external-cause mortality using Cox regression, both unadjusted and adjusted. Model 1 was adjusted for basic sociodemographics including age and sex. Model 2 was adjusted for expanded sociodemographics including age, sex, Aboriginal identity, marital status, IRSD, and remoteness. Model 3 was maximally adjusted using model 2 covariates plus problematic drug use, problematic alcohol use, involuntary index admission, CCI score, and offense history. Model selection was theory-driven (see eAppendix 6 in Supplement 1). The statistical significance level (α) was prespecified at .05. Proportional hazards assumptions for all variables in all models were deemed reasonable by inspecting log-log survival plots and plots of scaled Schoenfeld residuals vs analysis time. Because the age-specific mortality rates indicated that age moderated associations of recent criminal sanction type with mortality, with different patterns in those aged 65 years or older vs younger, we restricted survival analyses to those younger than 65 years; among those 65 years or older, there were insufficient person-years and events in most exposure categories. Data were analyzed between February 2023 and April 2024 using SAS version 9.4 (SAS Institute) and STATA version 18 (StataCorp LLC).

## Results

We identified 83 071 adults (35 791 female [43.1%]; 21 208 aged 25-34 years [25.5%]; 7984 Aboriginal [9.6%]) with a psychosis-related hospital admission in NSW between July 2001 to November 2017 (median [IQR] follow up, 9.5 [4.8-14.2] years). Table 1 presents further baseline characteristics (see also eTables 1-4 in Supplement 1).

**Table 1.** Participant Characteristics by Recent (Past 2 Years) Criminal Sanction Type at Study Entry

Characteristic	Participants by sanction type, No. (%)					
	No sanction (n = 72 236)	Community (n = 5978)	Diversion (n = 1043)	Imprisonment		Total (N = 83 071)
				Current (n = 1136)	Prior (n = 2678)	
Sex						
Female or other^a^	33 623 (46.5)	1427 (23.9)	222 (21.3)	117 (10.3)	402 (15.0)	35 791 (43.1)
Male	38 613 (53.5)	4551 (76.1)	821 (78.7)	1019 (89.7)	2276 (85.0)	47 280 (56.9)
Age group, y						
18-24	14 102 (19.5)	1657 (27.7)	239 (22.9)	281 (24.7)	636 (23.7)	16 915 (20.4)
25-34	17 118 (23.7)	2096 (35.1)	354 (33.9)	474 (41.7)	1166 (43.5)	21 208 (25.5)
35-44	14 979 (20.7)	1479 (24.7)	267 (25.6)	259 (22.8)	660 (24.6)	17 644 (21.2)
45-54	10 982 (15.2)	571 (9.6)	122 (11.7)	91 (8.0)	187 (7.0)	11 953 (14.4)
55-64	6884 (9.5)	137 (2.3)	46 (4.4)	27 (2.4)	27 (1.0)	7121 (8.6)
≥65	8171 (11.3)	38 (0.6)	15 (1.4)	<5 (NS)	<5 (NS)	8230 (9.9)
Aboriginal and/or Torres Strait Islander identity						
Yes	5360 (7.4)	1226 (20.5)	175 (16.8)	356 (31.3)	867 (32.4)	7984 (9.6)
No	66 622 (92.2)	4752 (79.5)	868 (83.2)	780 (68.7)	1811 (67.6)	74 833 (90.1)
Missing or unknown	254 (0.4)	0	0	0	0	254 (0.3)
Residential Index of Relative Socioeconomic Disadvantage						
Least disadvantaged	28 399 (39.3)	1813 (30.3)	390 (37.4)	270 (23.8)	680 (25.4)	31 552 (38.0)
Moderately disadvantaged	22 145 (30.7)	1962 (32.8)	289 (27.7)	117 (10.3)	902 (33.7)	25 415 (30.6)
Most disadvantaged	17 877 (24.7)	1825 (30.5)	283 (27.1)	735 (64.7)	910 (34.0)	21 630 (26.0)
Interstate resident	2164 (3.0)	75 (1.3)	22 (2.1)	6 (0.5)	16 (0.6)	2283 (2.7)
Missing or unknown	1651 (2.3)	303 (5.1)	59 (5.7)	8 (0.7)	170 (6.3)	2191 (2.6)
Residential remoteness						
Major cities	51 173 (70.8)	3854 (64.5)	781 (74.9)	1005 (88.5)	1739 (64.9)	58 552 (70.5)
Inner regional	13 490 (18.7)	1279 (21.4)	149 (14.3)	100 (8.8)	551 (20.6)	15 569 (18.7)
Outer regional or remote	3758 (5.2)	467 (7.8)	32 (3.1)	17 (1.5)	202 (7.5)	4476 (5.4)
Interstate resident	2164 (3.0)	75 (1.3)	22 (2.1)	6 (0.5)	16 (0.6)	2283 (2.7)
Missing or unknown	1651 (2.3)	303 (5.1)	59 (5.7)	8 (0.7)	170 (6.3)	2191 (2.6)
Married (including de facto)						
Yes	16 658 (23.1)	697 (11.7)	127 (12.2)	212 (18.7)	302 (11.3)	17 996 (21.7)
No	49 726 (68.8)	4792 (80.2)	805 (77.2)	830 (73.1)	2152 (80.4)	58 305 (70.2)
Missing or unknown	5852 (8.1)	489 (8.2)	111 (10.6)	94 (8.3)	224 (8.3)	6770 (8.1)
Problematic drug use	22 216 (30.8)	4304 (72.0)	557 (53.4)	652 (57.4)	2219 (82.9)	29 948 (36.1)
Problematic alcohol use	11 670 (16.2)	2277 (38.1)	315 (30.2)	307 (27)	1155 (43.1)	15 724 (18.9)
Involuntary index admission	33 388 (46.2)	3071 (51.4)	681 (65.3)	232 (20.4)	1323 (49.4)	38 695 (46.6)
Charlson Comorbidity Index score (past year of age)						
0	69 646 (96.4)	5767 (96.5)	1008 (96.6)	1094 (96.3)	2556 (95.4)	80 071 (96.4)
1	1264 (1.7)	60 (1.0)	18 (1.7)	19 (1.7)	23 (0.9)	1384 (1.7)
≥2	1326 (1.8)	151 (2.5)	17 (1.6)	23 (2.0)	99 (3.7)	1616 (1.9)
Offense conviction history						
None	61 668 (85.4)	0	0	40 (3.5)	77 (2.9)	61 785 (74.4)
Nonviolent offenses only	4725 (6.5)	2413 (40.4)	269 (25.8)	134 (11.8)	402 (15.0)	7943 (9.6)
Violent offenses	5826 (8.1)	3565 (59.6)	774 (74.2)	962 (84.7)	2199 (82.1)	13 326 (16.0)
Unknown offenses only	17 (<0.0)	0	0	0	0	17 (<0.0)
Died	10 335 (14.3)	541 (9.0)	122 (11.7)	91 (8.0)	266 (9.9)	11 355 (13.7)

Abbreviation: NS, not shown (due to corresponding number of participants being <5).

^a^ Other category not reported separately due to having less than 5 participants in the entire cohort; other included intersex.

Of the entire cohort, 25 824 (31.1%) experienced 1 or more recent criminal sanctions or mental health court diversions during follow up. Of all participants, 8953 (10.8%) were imprisoned and 8083 (9.7%) received mental health court diversion. Of those diverted, 5193 (64.3%) received a community sanction and 2595 (32.1%) were imprisoned before or after diversion.

### Cause of Death

A total of 11 355 deaths were recorded (Table 2), of which 5790 were among participants aged less than 65 years and 5565 were among those aged 65 years or older. In those 65 years or older, 5241 deaths (94.2%) were disease-related compared with 3041 deaths (52.5%) in those aged younger than 65 years. In people younger than 65 years, disease-related causes were the leading cause of death in those with no recent criminal sanction (2736 of 4692 deaths [58.3%]) and those with a recent community sanction (173 of 550 deaths [31.5%]), while suicide was the leading cause in those with recent mental health court diversion (64 of 189 deaths [33.9%]) and those currently imprisoned (13 of 25 deaths [52.0%]); accidental drug overdose was the leading cause in those with recent prior imprisonment (151 of 334 deaths [45.2%]).

**Table 2.** Cause of Death Category by Recent (Past 2 Years) Criminal Sanction Type at Death^a^

Cause of death by age group	Participant deaths by sanction type, No./total No. (%)					
	No sanction	Community	Diversion	Imprisonment		Total
				Current	Prior	
Age <65 y						
Disease-related	2736/4692 (58.3)	173/550 (31.5)	52/189 (27.5)	8/25 (32.0)	72/334 (21.6)	3041/5790 (52.5)
Suicide	1030/4692 (22.0)	131/550 (23.8)	64/189 (33.9)	13/25 (52.0)	66/334 (19.8)	1304/5790 (22.5)
Accidental drug overdose	509/4692 (10.8)	157/550 (28.5)	43/189 (22.8)	<5 (NS)	151/334 (45.2)	862/5790 (14.9)
Other external causes	294/4692 (6.3)	71/550 (12.9)	20/189 (10.6)	<5 (NS)	37/334 (11.1)	424/5790 (7.3)
Undefined or unknown	123/4692 (2.6)	18/550 (3.3)	10/189 (5.3)	0	8/334 (2.4)	159/5790 (2.7)
All external causes	1833/4692 (39.1)	359/550 (65.3)	127/189 (67.2)	17/25 (68.0)	254/334 (76.0)	2590/5790 (44.7)
Age ≥65 y						
Disease-related	5221/5540 (94.2)	10/11 (90.9)	7/11 (63.6)	<5 (NS)	<5 (NS)	5241/5565 (94.2)
Suicide	82/5540 (1.5)	0	<5 (NS)	0	0	85/5565 (1.5)
Accidental drug overdose	37/5540 (0.7)	0	<5 (NS)	0	0	38/5565 (0.7)
Other external causes	155/5540 (2.8)	0	0	0	0	155/5565 (2.8)
Undefined or unknown	45/5540 (0.8)	<5 (NS)	0	0	0	46/5565 (0.8)
All external causes	274/5540 (4.9)	0	<5 (NS)	0	0	278/5565 (5.0)
All ages						
Disease-related	7957/10 232 (77.8)	183/561 (32.6)	59/200 (29.5)	9/26 (34.6)	74/336 (22.0)	8282/11 355 (72.9)
Suicide	1112/10 232 (10.9)	131/561 (23.4)	67/200 (33.5)	13/26 (50.0)	66/336 (19.6)	1389/11 355 (12.2)
Accidental drug overdose	546/10 232 (5.3)	157/561 (28.0)	44/200 (22.0)	<5 (NS)	151/336 (44.9)	900/11 355 (7.9)
Other external causes	449/10 232 (4.4)	71/561 (12.7)	20/200 (10.0)	<5 (NS)	37/336 (11.0)	579/11 355 (5.1)
Undefined or unknown	168/10 232 (1.6)	19/561 (3.4)	10/200 (5.0)	0	8/336 (2.4)	205/11 355 (1.8)
All external causes	2107/10 232 (20.6)	359/561 (64.0)	131/200 (65.5)	17/26 (65.4)	254/336 (75.6)	2868/11 355 (25.3)

Abbreviation: NS, not shown (due to corresponding number of participants being <5).

^a^ Data source: Cause of Death Unit Record File held by the New South Wales Ministry of Health Secure Analytics for Population Health Research and Intelligence.

### All-Cause and External-Cause Mortality Rates

Age- and sex-specific all-cause mortality rates by recent criminal sanction type in those aged younger than 65 years are presented in eFigure 1 and eTable 5 in Supplement 1. Mortality rates tended to increase with age and be higher in males. In all age groups older than 24 years and younger than 65 years, all-cause mortality rates were highest in those with recent prior imprisonment. In all age groups younger than 65 years, all-cause mortality rates were lowest in those currently imprisoned, followed by those with no recent criminal sanction.

Age- and sex-specific external-cause mortality rates by recent criminal sanction type in those aged younger than 65 years are presented in eFigure 2 and eTable 6 in Supplement 1. External-cause mortality rates were more similar between age groups. In all age groups younger than 55 years, external-cause mortality rates were lowest in those currently imprisoned, followed by those with no recent criminal sanction. In all age groups older than 24 years and younger than 55 years, external-cause mortality rates were highest in those with recent prior imprisonment.

### Associations of Recent (Past 2 Years) Criminal Sanctions With Mortality

**Figure 2.**
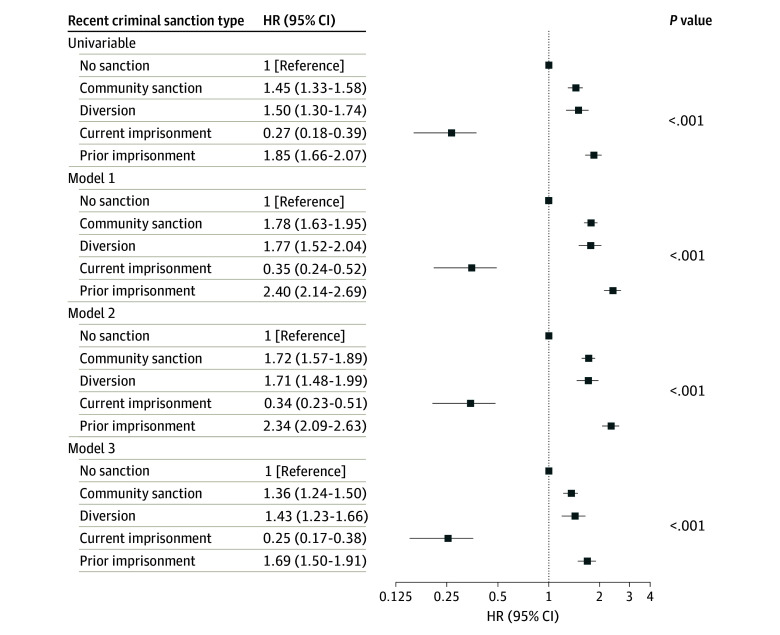
All-Cause Mortality by Recent (Past 2 Years) Criminal Sanction Type Among People With Psychosis Aged 18 to 64 Years (N = 74 841) Model 1 was adjusted for age and sex only. Model 2 was adjusted for model 1 covariates plus Aboriginal and/or Torres Strait Islander identity, marital status, residential Index of Relative Socioeconomic Disadvantage, and residential remoteness. Model 3 was adjusted for model 2 covariates plus history of problematic drug use, history of problematic alcohol use, involuntary index admission, Charlson Comorbidity Index score, and offense history. Error bars represent 95% CIs. HR indicates hazard ratio.

Figure 2 presents hazard ratios (HRs) showing associations of recent criminal sanction type with all-cause mortality in those aged younger than 65 years. In all models, compared with no recent criminal sanction, recent community sanction, mental health court diversion, and prior imprisonment were associated with increased hazards of all-cause mortality, including after adjustment for sociodemographic, health-related, and offense-related confounders. After maximal adjustment, the highest hazard of all-cause mortality was observed for recent prior imprisonment (model 3 adjusted HR [aHR], 1.69; 95% CI, 1.50-1.91), followed by mental health court diversion (aHR, 1.43; 95% CI, 1.23-1.66). Current imprisonment was associated with a lower hazard of all-cause mortality (aHR, 0.25; 95% CI, 0.17-0.38).

**Figure 3.**
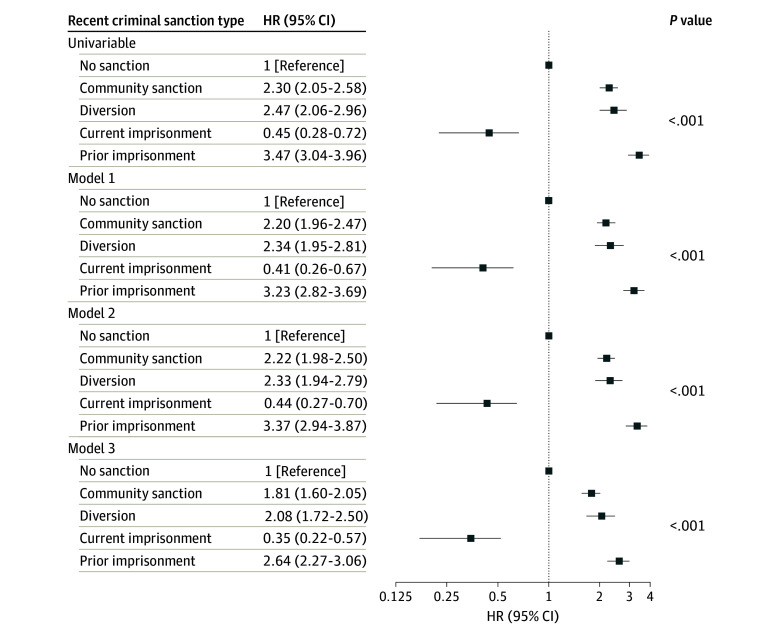
External-Cause Mortality by Recent (Past 2 Years) Criminal Sanction Type Among People With Psychosis Aged 18 to 64 Years (N = 74 841) Model 1 was adjusted for age and sex only. Model 2 was adjusted for model 1 covariates plus Aboriginal and/or Torres Strait Islander identity, marital status, residential Index of Relative Socioeconomic Disadvantage, and residential remoteness. Model 3 was adjusted for model 2 covariates plus history of problematic drug use, history of problematic alcohol use, involuntary index admission, Charlson Comorbidity Index score, offense history. Error bars represent 95% CIs. HR indicates hazard ratio.

Figure 3 presents HRs for associations of recent criminal sanction types with external-cause mortality in those younger than 65 years. Results were similar to those of all cause mortality, but generally of higher magnitude; all recent criminal sanction types other than current imprisonment were associated with increased hazards of external-cause mortality compared with no recent criminal sanction in all models, while current imprisonment was associated with a reduced hazard. Again, recent prior imprisonment was associated with the highest hazard of external-cause mortality (model 3 aHR, 2.64; 95% CI, 2.27-3.06), followed by mental health court diversion (aHR, 2.08; 95% CI, 1.72-2.50). Sex-stratified results were similar to those for both sexes combined (see eTables 7-10 in Supplement 1).

## Discussion

In this cohort study of adults hospitalized for psychosis in NSW, almost one-third received a criminal sanction or diversion (excluding for minor traffic offenses), and 1 in 10 were imprisoned. While there is no directly comparable data, this finding appears elevated compared with the NSW community.^32^ Whereas most deaths in those with no recent criminal sanction were disease-related, external causes—primarily suicide and accidental drug overdose—caused the majority of deaths in those with recent criminal sanctions. Among those aged younger than 65 years, compared with no recent criminal sanction, we found consistent associations of recent (past 2 years) mental health court diversion, community sanctions, and prior imprisonment with increased all-cause and external-cause mortality, independent of sociodemographic and health-related confounders and baseline offense history. To our knowledge, our study is the first among people with psychosis or SMI examining variation in mortality by criminal sanction type, including diversion.

Our findings concur with associations of CLS involvement with mortality documented in broader populations,^5,6,21^ including NSW,^7^ and several SMI cohorts.^8,9,10,11,12^ Unlike some Nordic studies,^9,10,11,12,33^ our results did not differ by sex, and our findings diverge from a Danish study^13^ reporting no association of forensic sentencing with mortality; this could be explained by differing mental health and legal systems,^14^ varying confounder adjustment, and/or baseline mortality differences within each study population. A novel finding of our study is that elevated mortality hazards extended to those receiving mental health court diversion, highlighting the need to consider this group in mortality prevention efforts. The reduced hazard of mortality associated with current imprisonment in our study has been documented in mainstream prison populations,^21,22,34^ likely reflecting the highly controlled prison environment; however, preventable deaths continue. Most were due to suicide in our study, reinforcing the importance of addressing factors contributing to prison suicide and avoiding imprisonment of people with psychosis.^35^

Pathways between psychosis, criminalized behavior, criminal sanctions, and mortality are complex and likely influenced by pre-existing factors such as psychosis severity, co-occurring substance use, treatment engagement, and socioeconomic exclusion.^36,37^ Criminal sanctions can also cause material and psychological harms,^36,38,39,40^ which may be exacerbated for people with psychosis,^3,41^ particularly those with intersecting experiences of multilayered discrimination and trauma, including Aboriginal and other racialized people^39,41,42,43^ and women.^39,42^ Aboriginal people were overrepresented in our cohort (9.6% vs 3.4% of NSW adults^44^), and within all criminal sanction categories, reflecting the nexus between adverse mental health outcomes and extreme overincarceration of Aboriginal people in Australia.^45^ These are recognized as being associated with systemic and institutional racism and colonial policies and practices, including land dispossession, frontier policing, cultural destruction, and forced child removals,^45,46^ highlighting the need to decolonize service systems and support Aboriginal-controlled responses.^47^

Despite its therapeutic intent, there are several reasons that mortality may remain elevated following CLS diversion, including the aforementioned pre-existing factors; reliance on mainstream community mental health services, private psychiatrists, and/or primary care clinicians,^18^ which may have limited resources or experience managing complex forensically involved patients; treatment disengagement^48^; and failure to address wider health determinants. Diversion may also be perceived punitively^49^ and engender similar stressors to traditional sanctions, including stigma and fear of imprisonment.^36,38^

Our results suggest a need for comprehensive cross-sectoral approaches to address overlapping clinical, social, and systemic determinants of CLS involvement and mortality in people with psychosis, particularly due to accidental overdose and suicide, and reduce harms of criminal sanctions. Increased resourcing of general community and specialist forensic mental health services, clarification of optimal models of integration between the two,^50^ and integrated treatment for co-occurring harmful substance use^51^ would likely assist. Future research should evaluate mortality, health, and person-centered outcomes associated with different forensic mental health service and legislative approaches and develop effective interventions and care models to optimize health and safety in people with psychosis involved in CLSs. More profoundly, abolition advocates propose a radical reconsideration of justice,^39,42^ a challenge the medical field must engage with.^37,40^

### Strengths and Limitations

Strengths of our study include the large population-based sample, time-varying assessment of criminal sanctions, and robust adjustment for confounders including sociodemographic factors, problematic drug and alcohol use, physical comorbidity, and offense history. Limitations include the observational design preventing causal inference and our inability to adjust for all potential confounders, such as individual socioeconomic status and pharmacotherapy. Our exposure classification simplified complex CLS interactions, and we did not examine police contacts, juvenile sanctions, or non–mental health diversion (eg, drug courts). We could not identify exposures, covariates, or deaths occurring interstate, overseas, or before the study period, although we expect these are minor. Finally, linkage and clerical errors may affect any probabilistic data-linkage study; however, the linkage method had a low (<0.5%) false-positive rate, and we excluded participants with suspected linkage error.

## Conclusions

In this cohort study of adults with psychosis, CLS involvement was common, with almost 1 in 3 individuals receiving a criminal sanction or diversion. Recent (past 2 years) criminal sanctions, including mental health court diversion, were associated with increased hazards of all-cause and external-cause mortality among those who were not currently imprisoned, compared with no recent sanction. Effective approaches to improving health outcomes among people with psychosis following CLS involvement should be developed.

## Notes

Supplement 1. **eAppendix 1.** Criteria for Identifying Cases of False-Positive Linkage Error **eAppendix 2.** Criteria for Excluding Individual Records due to Administrative Error **eAppendix 3.** Further Detail Regarding Section 32 and Section 33 Dismissals **eAppendix 4.** Further Detail Regarding Exposure Classification **eAppendix 5.** Systematized Nomenclature for Medicine: Clinical Terminology, Australian Release (SNOMED-CT-AU) Codes Used to Identify Problematic Drug and Alcohol Use **eAppendix 6.** Further Detail Regarding Covariate and Model Selection **eTable 1.** Participant Characteristics by Recent (Past 2 Years) Criminal Sanction Type at Study Entry for Participants Aged <65 Years at Entry **eTable 2.** Participant Characteristics by Recent (Past 2 Years) Criminal Sanction Type at Study Entry for Participants Aged ≥65 Years at Entry **eTable 3.** Participant Characteristics by Recent (Past 2 Years) Criminal Sanction Type at Last Observation Aged <65 Years **eTable 4.** Participant Characteristics by Recent (Past 2 Years) Criminal Sanction Type at Last Observation Aged ≥65 Years **eFigure 1.** Age- and Sex-Specific All-Cause Mortality Rates Among People With Psychosis Aged 18 to 64 Years by Recent (Past 2-Years) Criminal Sanction Type (n=74,841) **eTable 5.** Age- and Sex-Specific All-Cause Mortality Rates by Recent (Past 2 Years) Criminal Sanction Type **eFigure 2.** Age- and Sex-Specific External-Cause Mortality Rates Among People With Psychosis Aged 18 to 64 Years by Recent (Past 2 Years) Criminal Sanction Type (n=74,841) **eTable 6.** Age- and Sex-Specific External-Cause Mortality Rates by Recent (Past 2 Years) Criminal Sanction Type **eTable 7.** All-Cause Mortality Hazard Ratios by Recent (Past 2 Years) Criminal Sanction Type Among Men With Psychosis Aged 18 to 64 Years (n=44,287) **eTable 8.** All-Cause Mortality Hazard Ratios by Recent (Past 2 Years) Criminal Sanction Type Among Women With Psychosis Aged 18 to 64 Years (n=30,554) **eTable 9.** External-Cause Mortality Hazard Ratios by Recent (Past 2 Years) Criminal Sanction Type Among Men With Psychosis Aged 18 to 64 Years (n=44,287) **eTable 10.** External-Cause Mortality Hazard Ratios by Recent (Past 2 Years) Criminal Sanction Type Among Women With Psychosis Aged 18 to 64 Years (n=30,554)
eReferences

Supplement 2.
Data Sharing Statement
