# Supplementary material for: Mortality, Criminal Sanctions, and Court Diversion in People With Psychosis
Source: JAMA Netw Open. 2024 Oct 31;7(10):e2442146. doi: 10.1001/jamanetworkopen.2024.42146 (PMC11528309; doi:10.1001/jamanetworkopen.2024.42146)
Supplement: Supplement 1. — eAppendix 1. Criteria for Identifying Cases of False-Positive Linkage Error eAppendix 2. Criteria for Excluding Individual Records due to Administrative Error eAppendix 3. Further Detail Regarding Section 32 and Section 33 Dismissals eAppendix 4. Further Detail Regarding Exposure Classification eAppendix 5. Systematized Nomenclature for Medicine: Clinical Terminology, Australian Release (SNOMED-CT-AU) Codes Used to Identify Problematic Drug and Alcohol Use eAppendix 6. Further Detail Regarding Covariate and Model Selection eTable 1. Participant Characteristics by Recent (Past 2 Years) Criminal Sanction Type at Study Entry for Participants Aged <65 Years at Entry eTable 2. Participant Characteristics by Recent (Past 2 Years) Criminal Sanction Type at Study Entry for Participants Aged ≥65 Years at Entry eTable 3. Participant Characteristics by Recent (Past 2 Years) Criminal Sanction Type at Last Observation Aged <65 Years eTable 4. Participant Characteristics by Recent (Past 2 Years) Criminal Sanction Type at Last Observation Aged ≥65 Years eFigure 1. Age- and Sex-Specific All-Cause Mortality Rates Among People With Psychosis Aged 18 to 64 Years by Recent (Past 2-Years) Criminal Sanction Type (n=74,841) eTable 5. Age- and Sex-Specific All-Cause Mortality Rates by Recent (Past 2 Years) Criminal Sanction Type eFigure 2. Age- and Sex-Specific External-Cause Mortality Rates Among People With Psychosis Aged 18 to 64 Years by Recent (Past 2 Years) Criminal Sanction Type (n=74,841) eTable 6. Age- and Sex-Specific External-Cause Mortality Rates by Recent (Past 2 Years) Criminal Sanction Type eTable 7. All-Cause Mortality Hazard Ratios by Recent (Past 2 Years) Criminal Sanction Type Among Men With Psychosis Aged 18 to 64 Years (n=44,287) eTable 8. All-Cause Mortality Hazard Ratios by Recent (Past 2 Years) Criminal Sanction Type Among Women With Psychosis Aged 18 to 64 Years (n=30,554) eTable 9. External-Cause Mortality Hazard Ratios by Recent (Past 2 Years) Criminal Sanction T [file jamanetwopen-e2442146-s001.pdf]

Supplemental Online Content

Spike E, Srasuebkul P, Butler T, et al. Mortality, criminal sanctions, and court diversion in people with psychosis. *JAMA Netw. Open*. 2024;7(10):e2442146. doi:10.1001/jamanetworkopen.2024.42146

eAppendix 1. Criteria for Identifying Cases of False-Positive Linkage Error
eAppendix 2. Criteria for Excluding Individual Records due to Administrative Error
eAppendix 3. Further Detail Regarding Section 32 and Section 33 Dismissals
eAppendix 4. Further Detail Regarding Exposure Classification
eAppendix 5. Systematized Nomenclature for Medicine–Clinical Terminology, Australian Release (SNOMED-CT-AU) Codes Used To Identify Problematic Drug and Alcohol Use
eAppendix 6. Further Detail Regarding Covariate And Model Selection
eTable 1. Participant Characteristics by Recent (Past 2 Years) Criminal Sanction Type at Study Entry for Participants Aged <65 Years at Entry
eTable 2. Participant Characteristics by Recent (Past 2 Years) Criminal Sanction Type at Study Entry for Participants Aged ≥65 Years at Entry
eTable 3. Participant Characteristics by Recent (Past 2 Years) Criminal Sanction Type at Last Observation Aged <65 Years
eTable 4. Participant Characteristics by Recent (Past 2 Years) Criminal Sanction Type at Last Observation Aged ≥65 Years
eFigure 1. Age- and Sex-Specific All-Cause Mortality Rates Among People With Psychosis Aged 18 to 64 Years by Recent (Past 2 Years) Criminal Sanction Type (n=74,841)
eTable 5. Age- and Sex-Specific All-Cause Mortality Rates by Recent (Past 2 Years) Criminal Sanction Type
eFigure 2. Age- and Sex-Specific External-Cause Mortality Rates Among People With Psychosis Aged 18 to 64 Years by Recent (Past 2 Years) Criminal Sanction Type (n=74,841)
eTable 6. Age- and Sex-Specific External-Cause Mortality Rates by Recent (Past 2 Years) Criminal Sanction Type
eTable 7. All-Cause Mortality Hazard Ratios by Recent (Past 2 Years) Criminal Sanction Type Among Men With Psychosis Aged 18 to 64 Years (n=44,287)
eTable 8. All-Cause Mortality Hazard Ratios by Recent (Past 2 Years) Criminal Sanction Type Among Women With Psychosis Aged 18 to 64 Years (n=30,554)
eTable 9. External-Cause Mortality Hazard Ratios by Recent (Past 2 Years) Criminal Sanction Type Among Men With Psychosis Aged 18 to 64 Years (n=44,287)
eTable 10. External-Cause Mortality Hazard Ratios by Recent (Past 2 Years) Criminal Sanction Type Among Women With Psychosis Aged 18 to 64 Years (n=30,554)
eReferences

This supplemental material has been provided by the authors to give readers additional information about their work.

© 2024 Spike E et al. *JAMA Network Open*

eAppendix 1. Criteria for Identifying Cases of False-Positive Linkage Error

Criteria for identifying cases of possible linkage error are outlined below. If any participant met any of the below criteria, it was considered likely that the participant had one or more false positive linkage errors and/or that key data was unreliable, and the participant was excluded from the study.

**Dataset**	**Criteria for identifying linkage error**
Admitted Patient Data Collection (APDC)	• Birth month and year after month and year of episode start date or episode end date<br>• Age >116 years at episode start date or episode end date<br>• Date of death >3 days before episode start date<br>• Date of death >28 days before episode end date
Emergency Department Data Collection (EDDC)	• Birth month and year after month and year of arrival date or departure date<br>• Age >116 years at arrival date or departure date<br>• Mode of separation is not "dead on arrival" and death date is >3 days before arrival date<br>• Mode of separation is not "dead on arrival" and death date is >6 days before departure date<br>• Mode of separation is "dead on arrival" and death date is >365 days before arrival date<br>• Mode of separation is "dead on arrival" and death date is >368 days before departure date
Re-offending Database (RoD)	• Age <7 years at offense date or finalization date<br>• Age >116 years at offense date or finalization date<br>• Death date is earlier than offense date<br>• Death date is earlier than finalization date, and both appearance-level outcome and charge-level outcomes are not consistent with death (i.e. appearance-level outcome is not among "All charges withdrawn by prosecution", "Convicted ex-parte", "Otherwise disposed of (e.g. transferred to drug court, deceased)" and charge-level outcome is not among "Otherwise disposed of", "Withdrawn", "Unknown", missing).
Offender Integrated Management System (OIMS)	• Age <7 years at episode start date or episode end date<br>• Age >116 years at episode start date or episode end date<br>• Death date < episode start date<br>• Death date >3 days before episode end date<br>• Episode start or end dates overlap by >1 day<br>• Variable containing number of prior episodes is not consecutive when episodes are ordered chronologically
Registry of Births, Deaths and Marriages Deaths Registrations (RBDM-DR) and Australian Co-ordinating Registry Cause of Death Unit Record File (COD-URF)	• Multiple death dates with a discrepancy of >14 days either between or within mortality datasets<br>• Birth date later than death date<br>• Age >116 years at death

eAppendix 2. Criteria for Excluding Individual Records due to Administrative Error

Criteria for exclusion of individual records within each study dataset are outlined below. Date ranges were selected based either on extremes of plausibility, file creation dates, or methodological information provided to the researchers by the NSW Centre for Health Record Linkage. In addition, as follow up for this study ended on 31 May 2019, records in all datasets were censored on this date.

**Dataset**	**Criteria for exclusion of individual records due to administrative error**
Admitted Patient Data Collection (APDC)	• Episode start date <01 July 1886, >31 March 2021, or missing<br>• Episode end date <01 July 2001, >31 March 2021, or missing<br>• Episode start date after episode end date<br>• Mode of separation identified as "registered in error"
Emergency Department Data Collection (EDDC)	• Arrival date <01 Jan 2005, >31 March 2021, or missing<br>• Departure date <01 Jan 2005 or >24 March 2022<br>• Arrival date after departure date<br>• Calculated length of stay in Emergency Department >7 days<br>• Mode of separation identified as "registered in error"<br><br>*Note: missing departure dates in the EDDC were imputed with the arrival date as length of stay in the Emergency Department is usually <24 hours*.
Re-offending Database (RoD)	• Charge finalization date <01 July 2001, > 31 December 2020, or missing<br>• Offense date <01 July 1901 or >31 December 2020<br>• Offense date after charge finalization date<br><br>*Note: missing offense dates in the RoD were imputed with the charge finalization date as the offense date must logically precede the finalization date.*
Offender Integrated Management System (OIMS)	• Episode start date <01 July 1895, >20 April 2022, or missing<br>• Episode end date <01 July 2001 or >20 April 2022<br>• Episode start date after episode end date<br><br>*Note: missing episode end dates in OIMS indicate current imprisonment at the time of data extraction.*
Registry of Births, Deaths and Marriages Deaths Registrations (RBDM-DR) and Australian Co-ordinating Registry Cause of Death Unit Record File (COD-URF)	• Date of death <01 July 2001, >31 March 2021, or missing in both datasets<br><br>*Note: Where multiple dates of death were recorded for a given participant in one or both datasets, and the discrepancy between all death dates was ≤14 days, then the earliest date of death recorded in the RBDM-DR was used; if no date of death was recorded in the RBDM-DR, the earliest date of death in the COD-URF was used.*

eAppendix 3. Further Detail Regarding Section 32 and Section 33 Dismissals

Section 32 and 33 of the NSW *Mental Health (Forensic Provisions) Act 1990*[1] provided diversion mechanisms for certain offenses on the basis of mental illness or cognitive impairment. The NSW *Mental Health (Forensic Provisions) Act 1990* was repealed in 2020 and replaced by the NSW *Mental Health and Cognitive Impairment Forensic Provisions Act 2020*,[2] which contains similar diversion provisions. Both Section 32 and Section 33 applied only to summary offenses and indictable offenses triable summarily, and in practice were almost exclusively used by the lower courts (i.e. the NSW Local Court and the NSW Children's Court).

Section 32 applied to defendants who were "(i) cognitively impaired, or (ii) suffering from mental illness, or (iii) suffering from a mental condition for which treatment is available in a mental health facility". Section 32(3) enabled a magistrate to dismiss a charge and discharge a defendant (a) into the care of a responsible person, unconditionally or subject to conditions, or (b) on the condition that the defendant attend on a person or at a place specified by the Magistrate: (i) for assessment or treatment (or both) of the defendant's mental condition or cognitive impairment, or (ii) to enable the provision of support in relation to the defendant's cognitive impairment, or (c) unconditionally." If a person did not comply with an order under Section 32(3) within 6 months of the order being made, they could be brought back before the court and dealt with as if they had not been discharged.

Section 33 applied to defendants who appeared to be "mentally ill" and enabled a magistrate to order that the defendant be detained in a mental health facility for assessment, and to be brought back before the court if not found to be a mentally ill or mentally disordered person.

eAppendix 4. Further Detail Regarding Exposure Classification

The main exposure of interest, recent (past two-year) adult criminal sanction type, was constructed as a categorical, time-varying variable (updated at daily intervals based on data from the two years preceding the ascertainment date) with five mutually exclusive levels: no recent criminal sanction, recent mental health court diversion, recent community sanction, current imprisonment, and recent prior imprisonment (i.e. recent release from prison). At cohort entry, ascertainment of recent adult criminal sanctions was extended retrospectively to the preceding two years where possible, however criminal sanction data was only available from 1 July 2001. Therefore, ascertainment of recent criminal sanctions may have been incomplete for participants who entered the study between 1 July 2001 and 1 July 2003.

A hierarchical classification was applied, such that current imprisonment overrode all other categories; prior imprisonment and mental health court diversion overrode community sanction; mental health court diversion overrode prior imprisonment if it was received *after* release from the most recent imprisonment; and prior imprisonment overrode mental health court diversion if release occurred *after* the most recent court diversion. The rationale for this approach is that it is common to receive multiple types of criminal sanctions within a short space of time, and we needed to construct an exposure variable which could capture this complexity. We did so *a priori*, with specific justifications for the choice of each category outlined below.

Recent (past two-year) criminal sanction type was a derived variable we constructed based on variables in the Offender Integrated Management System (OIMS) dataset and the Re-offending Database (RoD) as outlined below.

**Recent criminal sanction type**	**Definition and hierarchical classification**	**Variable/s (dataset) used for ascertainment**	**Justification for category**
No recent criminal sanction	No criminal sanction corresponding to any of the four other criminal sanction categories recorded for this participant in the two years prior to the ascertainment date	N/A	This category is designed to capture the impact of the absence of any significant criminal sanction in the past two years i.e. the baseline exposure category.
Recent community sanction	Participant received a court-ordered community penalty e.g. fine, supervised order in relation to an offense in the two years prior to the ascertainment date<br><br>AND<br><br>Participant did not receive a criminal sanction corresponding to the categories of mental health court diversion, current imprisonment or prior imprisonment in the two years prior to the ascertainment date	Penalty type (RoD)	This category is designed to capture the impact of receiving a court-ordered community sanction in the past two years. It includes a spectrum of penalties ranging from those that are relatively minor (e.g. conviction only, small fines) to those with significant supervision requirements and conditions (e.g. Intensive Correction Orders).
Recent mental health court diversion	Participant received mental health court diversion in the two years prior to the ascertainment date<br><br>Participant may also have received a community sanction at any time or been released from prison in the two years prior to the ascertainment date, as long as the release from imprisonment occurred *before* or on the date that they received mental health court diversion	Mental health dismissal type (RoD)	This category is designed to capture the impact of receiving mental health court diversion in the past two years. In practice, many people who receive court diversion are likely to have also received a community-based sanction or to have been imprisoned for a brief

**Recent criminal sanction type**	**Definition and hierarchical classification**	**Variable/s (dataset) used for ascertainment**	**Justification for category**
			period while awaiting court, and we would therefore expect any beneficial (or adverse) impacts of court diversion to persist in the presence of other community-based criminal sanctions, or imprisonment episodes that preceded the receipt of court diversion.
Current imprisonment	Participant is imprisoned in a NSW adult prison on the ascertainment date. Current imprisonment is considered to commence on episode start date of a given imprisonment episode and continue until the corresponding episode end date inclusive.<br><br>Participant may have received any other criminal sanction type in the two years prior to the ascertainment date	Episode start and end dates (OIMS)	This category is designed to capture the impact of current imprisonment. The unique and highly controlled nature of the prison environment is likely to outweigh the impacts of any other criminal sanctions and mortality during imprisonment is known to be substantially lower than after release from prison,[3,4] making it necessary to differentiate between current and prior imprisonment.
Recent prior imprisonment	Participant was released from a NSW adult prison in the two years prior to the ascertainment date<br><br>Participant may have received a community sanction at any time or received mental health court diversion in the two years prior to the ascertainment date, as long as mental health court diversion was received *before* the most recent episode of imprisonment and release from imprisonment	Episode start and end dates (OIMS)	This category is designed to capture the impact of prior imprisonment in the past two years. Given the well-documented association between release from imprisonment and mortality,[5] and the significant stressors faced upon community re-entry, we expected that this would be likely to outweigh the impacts of most other criminal sanctions, including previous court diversion, given that imprisonment is likely to cause a significant interruption to community-based mental health care initiated through court diversion.

Current and prior imprisonment were ascertained based on episode start and end dates in the OIMS dataset, with current imprisonment episodes commencing on the imprisonment episode start date and prior imprisonment commencing on the day after the episode end date. Recent community sanction and mental health court diversion were ascertained based on the variables 'Penalty type' and 'Mental health dismissal type' recorded in the RoD, as outlined below. Given our focus on adult sanctions, we excluded community sanctions and court diversions for offenses finalized in the NSW Children's Court or a Youth Justice Conference, or in any court prior to a person's 18th birthday, and juvenile-specific sanctions (i.e. Juvenile Control Orders and Juvenile Probation Orders) irrespective of age.

Variable name	Default classification of recent criminal sanction type	Variable values corresponding to default classification of recent criminal sanction type
**Penalty type**	**No criminal sanction**	No conviction recorded<br>No penalty<br>Non-court<br>Other penalties<br>Penalty unknown<br>Periodic detention*<br>Imprisonment*<br><br>**Current and prior imprisonment, including periodic detention, were based on actual episode start and end dates as recorded in OIMS, not on the penalty type recorded in RoD.*
	**Community sanction**	Bond with supervision<br>Bond without conviction with supervision<br>Bond without conviction without supervision<br>Bond without supervision<br>Community Correction Order with supervision<br>Community Correction Order without supervision<br>Conditional Release Order with conviction, with supervision<br>Conditional Release Order with conviction, without supervision<br>Conditional Release Order without conviction, with supervision<br>Conviction only<br>Fine<br>Home detention<br>Intensive Correction Order<br>Pre-reform Intensive Correction Order<br>Pre-reform or Children's Community Service Order<br>Suspended sentence with supervision<br>Suspended sentence without supervision
**Mental health dismissal type**	**No criminal sanction**	Other*<br><br>**This refers to other/unspecified forms of mental health dismissal including dismissal by the higher courts due to reason of mental illness. Due to the low numbers of participants who received these dismissals (n=616) and a lack of clarity about the nature of these mental health dismissals, these were not assigned to a separate exposure category and were instead included with the baseline category.*
	**Mental health court diversion**	S32<br>S33<br>S32/S33/other*<br><br>**Prior to 2005, this variable did not distinguish between Section 32 and Section 33 dismissals and other types of mental health dismissal. This value only applies to records finalized in the lower courts prior to 2005.*

eAppendix 5. Systematized Nomenclature for Medicine–Clinical Terminology, Australian Release (SNOMED-CT-AU) Codes Used to Identify Problematic Drug and Alcohol Use

SNOMED-CT-AU code	Description	Whether used to identify problematic drug use (d), alcohol use (a) or both (b)
231467000	Absinthe addiction	a
300939009	Abstinent alcoholic	a
712542001	Abstinent from drug misuse	d
712543006	Abstinent from drug misuse in normal environment	d
712544000	Abstinent from drug misuse in protected environment	d
712545004	Abstinent from drug misuse on maintenance replacement	d
712546003	Abstinent from drug misuse when receiving blocking therapy	d
268727002	Abuse of non-dependence-producing substances	d
105549004	Abuses volatile solvents	d
296292003	Accidental amphetamine overdose	d
295167001	Accidental buprenorphine overdose	d
290175009	Accidental buprenorphine poisoning	d
296302007	Accidental cannabis overdose	d
291246000	Accidental cannabis poisoning	d
242263000	Accidental exposure to alcohol	a
242265007	Accidental exposure to ethanol	a
295194002	Accidental fentanyl overdose	d
290202004	Accidental fentanyl poisoning	d
295175007	Accidental heroin overdose	d
242358002	Accidental ingestion of hallucinogenic mushrooms	d
295508004	Accidental ketamine overdose	d
290491001	Accidental ketamine poisoning	d
295567006	Accidental local cocaine overdose	d
295185008	Accidental morphine overdose	d
290220008	Accidental narcotic poisoning	d
296322006	Accidental overdose by cocaine	d
296326009	Accidental overdose by crack cocaine	d
296128004	Accidental overdose of benzodiazepine	d
242255001	Accidental overdose of non-barbiturate hypnotic	d
297199006	Accidental overdose of opiate	d
269765000	Accidental poisoning by alcohol	a
216633005	Accidental poisoning by alcoholic beverage	a
216559001	Accidental poisoning by amphetamine	d
216530001	Accidental poisoning by benzodiazepine-based tranquilizer	d
216551003	Accidental poisoning by cannabis derivatives	d
216562003	Accidental poisoning by central nervous system stimulants	d
216583009	Accidental poisoning by cocaine	d
291261004	Accidental poisoning by crack cocaine	d

SNOMED-CT-AU code	Description	Whether used to identify problematic drug use (d), alcohol use (a) or both (b)
216635003	Accidental poisoning by denatured alcohol	a
216651006	Accidental poisoning by fusel oil	a
216550002	Accidental poisoning by hallucinogens	d
216463005	Accidental poisoning by heroin	d
216645001	Accidental poisoning by isopropyl alcohol	a
216640006	Accidental poisoning by methyl alcohol	a
216468001	Accidental poisoning by morphine	d
432353006	Accidental poisoning by opiate agonist	d
216469009	Accidental poisoning by opium	d
216558009	Accidental poisoning by psychostimulants	d
216648004	Accidental poisoning by rubbing alcohol substitute	a
699012001	Accidental poisoning by sedative	d
287166006	Accidental poisoning with ethyl alcohol	a
231482005	Active drug dependence	d
191802004	Acute alcoholic intoxication in alcoholism	a
191806001	Acute alcoholic intoxication in remission, in alcoholism	a
231466009	Acute drug intoxication	d
699124006	Admission to substance misuse detoxification center	d
704197006	Admits alcohol use	a
183486001	Admitted to alcohol detoxification center	a
705132005	Admitted to substance misuse detoxification center	d
218791008	Adverse reaction to cannabis	d
218790009	Adverse reaction to hallucinogen	d
218792001	Adverse reaction to lysergide	d
53041004	Alcohol	a
15167005	Alcohol abuse	a
440652002	Alcohol abuse cessation behavior	a
737363002	Alcohol abuse surveillance	a
73097000	Alcohol amnestic disorder	a
429291000124102	Alcohol brief intervention	a
429391000124108	Alcohol brief intervention refused	a
413473000	Alcohol consumption counseling	a
427013000	Alcohol consumption during pregnancy	a
66590003	Alcohol dependence	a
10755041000119100	Alcohol dependence in childbirth	a
10741871000119100	Alcohol dependence in pregnancy	a
413130000	Alcohol disorder monitoring	a
7052005	Alcohol hallucinosis	a
720175009	Alcohol harm reduction program	a
288031000119105	Alcohol induced disorder co-occurrent and due to alcohol dependence	a
417633001	Alcohol induced hallucinations	a

SNOMED-CT-AU code	Description	Whether used to identify problematic drug use (d), alcohol use (a) or both (b)
160573003	Alcohol intake	a
365967005	Alcohol intake - finding	a
160592001	Alcohol intake above recommended sensible limits	a
429775004	Alcohol intake exceeds recommended daily limit	a
102612005	Alcohol intolerance	a
25702006	Alcohol intoxication	a
18653004	Alcohol intoxication delirium	a
707166002	Alcohol reduction program	a
35637008	Alcohol rehabilitation	a
20093000	Alcohol rehabilitation and detoxification	a
720176005	Alcohol relapse prevention program	a
720178006	Alcohol twelve step program	a
228956008	Alcohol units	a
8635005	Alcohol withdrawal delirium	a
191476005	Alcohol withdrawal hallucinosis	a
191480000	Alcohol withdrawal syndrome	a
308742005	Alcohol withdrawal-induced convulsion	a
333164008	Alcohol-containing product	a
34938008	Alcohol-induced anxiety disorder	a
53936005	Alcohol-induced mood disorder	a
29212009	Alcohol-induced organic mental disorder	a
42344001	Alcohol-induced psychosis	a
61144001	Alcohol-induced psychotic disorder with delusions	a
78524005	Alcohol-induced sexual dysfunction	a
41083005	Alcohol-induced sleep disorder	a
361267005	Alcohol-related fit	a
53527002	Alcoholic beverage	a
228317009	Alcoholic binges exceeding safe amounts	a
228316000	Alcoholic binges exceeding sensible amounts	a
230800004	Alcoholic coma	a
192811002	Alcoholic encephalopathy	a
191478006	Alcoholic paranoia	a
7200002	Alcoholism	a
24165007	Alcoholism counseling	a
274517002	Alcoholism detoxication center	a
32663005	Allyl alcohol	a
55816008	Amino alcohol	a
724687008	Amnestic disorder caused by anxiolytic	d
724686004	Amnestic disorder caused by hypnotic	d
724685000	Amnestic disorder caused by sedative	d
703842006	Amphetamine	d
84758004	Amphetamine abuse	d
427205009	Amphetamine abuse, continuous	d

© 2024 Spike E et al. *JAMA Network Open*

SNOMED-CT-AU code	Description	Whether used to identify problematic drug use (d), alcohol use (a) or both (b)
429692000	Amphetamine abuse, episodic	d
412035000	Amphetamine and amphetamine derivative	d
774531008	Amphetamine and dextroamphetamine only product	d
116344009	Amphetamine aspartate	d
8837000	Amphetamine delirium	d
32358001	Amphetamine delusional disorder	d
21647008	Amphetamine dependence	d
275773002	Amphetamine in urine	d
434971000124109	Amphetamine induced psychotic disorder	d
45421006	Amphetamine intoxication	d
428659002	Amphetamine misuse	d
191845006	Amphetamine or psychostimulant dependence in remission	d
191843004	Amphetamine or psychostimulant dependence, continuous	d
191844005	Amphetamine or psychostimulant dependence, episodic	d
296291005	Amphetamine overdose	d
296294002	Amphetamine overdose of undetermined intent	d
291242003	Amphetamine poisoning of undetermined intent	d
87148003	Amphetamine sulfate	d
78358001	Amphetamine withdrawal	d
82339009	Amphetamine-induced anxiety disorder	d
43497001	Amphetamine-induced mood disorder	d
83367009	Amphetamine-induced organic mental disorder	d
51443000	Amphetamine-induced psychotic disorder with hallucinations	d
70932007	Amphetamine-induced sexual dysfunction	d
25753007	Amphetamine-induced sleep disorder	d
724723002	Anxiety disorder caused by ketamine	d
724708007	Anxiety disorder caused by methylenedioxymethamphetamine	d
762331007	Anxiety disorder caused by stimulant	d
737341006	Anxiety disorder caused by synthetic cannabinoid	d
231470001	Anxiolytic dependence	d
724659004	Anxiolytic dependence with current use	d
361148000	Anxiolytic intoxication	d
724667007	Anxiolytic substance withdrawal with perceptual disturbances	d
724661008	Anxiolytic withdrawal	d
724664000	Anxiolytic withdrawal without complication	d
312936002	Anxiolytic-induced organic mental disorder	d
713127001	Assessment of alcohol use	a
428211000124100	Assessment of substance use	d
711008001	Assessment of substance withdrawal	d
445628007	Assessment using alcohol withdrawal scale	a

© 2024 Spike E et al. *JAMA Network Open*

SNOMED-CT-AU code	Description	Whether used to identify problematic drug use (d), alcohol use (a) or both (b)
183388004	Aversion therapy - alcoholism	a
231472009	Barbiturate dependence	d
428623008	Barbiturate misuse	d
372615004	Barbiturate sedative	d
292337007	Barbiturate sedative adverse reaction	d
425339005	Barbiturate withdrawal	d
231473004	Benzodiazepine dependence	d
712742003	Benzodiazepine intoxication	d
428406005	Benzodiazepine misuse	d
296053004	Benzodiazepine overdose	d
703849002	Benzodiazepine withdrawal	d
703850002	Benzodiazepine withdrawal delirium	d
703847000	Buprenorphine + naloxone withdrawal	d
703845008	Buprenorphine dependence	d
295166005	Buprenorphine overdose	d
295169003	Buprenorphine overdose of undetermined intent	d
290174008	Buprenorphine poisoning	d
290177001	Buprenorphine poisoning of undetermined intent	d
703846009	Buprenorphine withdrawal	d
78723001	Cannabinosis	d
22924007	Cannabis	d
398705004	Cannabis	d
37344009	Cannabis abuse	d
63649001	Cannabis delusional disorder	d
85005007	Cannabis dependence	d
191839003	Cannabis dependence in remission	d
723933008	Cannabis dependence with current use	d
191837001	Cannabis dependence, continuous	d
191838006	Cannabis dependence, episodic	d
11047881000119100	Cannabis hyperemesis syndrome co-occurrent and due to cannabis abuse	d
11048011000119100	Cannabis hyperemesis syndrome co-occurrent and due to cannabis dependence	d
23527004	Cannabis intoxication	d
39807006	Cannabis intoxication delirium	d
763661003	Cannabis leaf	d
428823006	Cannabis misuse	d
296301000	Cannabis overdose	d
296304008	Cannabis overdose of undetermined intent	d
291248004	Cannabis poisoning of undetermined intent	d
10083006	Cannabis sativa	d
16292008	Cannabis sativa poisoning	d
703848005	Cannabis withdrawal	d
39951001	Cannabis-induced anxiety disorder	d
77355000	Cannabis-induced organic mental disorder	d

© 2024 Spike E et al. *JAMA Network Open*

SNOMED-CT-AU code	Description	Whether used to identify problematic drug use (d), alcohol use (a) or both (b)
26714005	Cannabis-induced psychotic disorder with hallucinations	d
296290006	Central nervous system stimulant overdose	d
228355004	Charged with drunk driving offence	a
191475009	Chronic alcoholic brain syndrome	a
191813001	Chronic alcoholism in remission	a
135331000119105	Circadian rhythm sleep disorder caused by alcohol	a
387085005	Cocaine	d
78267003	Cocaine abuse	d
70328006	Cocaine delirium	d
30491001	Cocaine delusional disorder	d
31956009	Cocaine dependence	d
191833002	Cocaine dependence in remission	d
724688003	Cocaine dependence with current use	d
191831000	Cocaine dependence, continuous	d
191832007	Cocaine dependence, episodic	d
27956007	Cocaine intoxication	d
409894009	Cocaine metabolite	d
429782000	Cocaine misuse	d
290545007	Cocaine poisoning of undetermined intent	d
733461000	Cocaine user	d
80868005	Cocaine withdrawal	d
51493001	Cocaine-induced anxiety disorder	d
10327003	Cocaine-induced mood disorder	d
46975003	Cocaine-induced organic mental disorder	d
6348008	Cocaine-induced psychotic disorder with hallucinations	d
8686000	Cocaine-induced sexual dysfunction	d
22574000	Cocaine-induced sleep disorder	d
87106005	Combined alcohol and drug detoxification	b
62213004	Combined alcohol and drug rehabilitation	b
23915005	Combined alcohol and drug rehabilitation and detoxification	b
429299000	Combined drug dependence, continuous	d
191873008	Combined drug dependence, excluding opioid, continuous	d
191874002	Combined drug dependence, excluding opioid, episodic	d
191875001	Combined drug dependence, excluding opioid, in remission	d
191871005	Combined drug dependence, excluding opioids	d
191865004	Combined opioid with other drug dependence	d
191869005	Combined opioid with other drug dependence in remission	d
191867007	Combined opioid with other drug dependence, continuous	d

SNOMED-CT-AU code	Description	Whether used to identify problematic drug use (d), alcohol use (a) or both (b)
191868002	Combined opioid with other drug dependence, episodic	d
413897002	Community detoxification registered	d
191804003	Continuous acute alcoholic intoxication in alcoholism	a
191811004	Continuous chronic alcoholism	a
191819002	Continuous opioid dependence	d
125851000119106	Continuous sedative abuse	d
231481003	Controlled drug dependence	d
228356003	Convicted of drunk driving	a
428493006	Crack cocaine misuse	d
228365005	Craving for alcohol	a
219006	Current drinker	a
228319007	Daily drinker	a
133301000119102	Degenerative brain disorder caused by alcohol	a
724716003	Delirium caused by ketamine	d
724705005	Delirium caused by methylenedioxymethamphetamine	d
762324008	Delirium caused by stimulant	d
737339005	Delirium caused by synthetic cannabinoid	d
281004	Dementia associated with alcoholism	a
724714000	Dependence caused by dissociative substance	d
724715004	Dependence caused by ketamine	d
441681009	Dependence on unknown drug	d
6525002	Dependent drug abuse	d
182969009	Dependent drug detoxification	d
363905002	Details of alcohol drinking behavior	a
363908000	Details of drug misuse behavior	d
64297001	Detoxication psychiatric therapy for alcoholism	a
67516001	Detoxification therapy	d
231474005	Diazepam dependence	d
413989008	Did not attend substance misuse clinic	d
228326007	Drinking binge	a
228312003	Drinks alcohol evenly through week	a
228313008	Drinks alcohol unevenly through week	a
447087000	Drinks alcoholic cider	a
228373001	Drug addict	d
185095001	Drug addict re-notific due	d
60112009	Drug addiction counseling	d
270393005	Drug addiction notification	d
310369001	Drug addiction notification status	d
266707007	Drug addiction therapy	d
310653000	Drug addiction therapy - methadone	d
191816009	Drug dependence	d
199252002	Drug dependence during pregnancy - baby delivered	d

SNOMED-CT-AU code	Description	Whether used to identify problematic drug use (d), alcohol use (a) or both (b)
199254001	Drug dependence during pregnancy - baby not yet delivered	d
267206008	Drug dependence during pregnancy, childbirth and the puerperium	d
414054004	Drug dependence home detoxification	d
414055003	Drug dependence home detoxification contraindicated	d
10755161000119100	Drug dependence in childbirth	d
34150001	Drug dependence in mother complicating pregnancy, childbirth AND/OR puerperium	d
1461000119109	Drug dependence in remission	d
199253007	Drug dependence in the puerperium - baby delivered	d
199255000	Drug dependence in the puerperium - baby delivered during previous episode of care	d
414056002	Drug dependence self detoxification	d
153491000119103	Drug dependence, continuous	d
153501000119105	Drug dependence, episodic	d
61480009	Drug detoxification	d
56876005	Drug rehabilitation and detoxification	d
363101005	Drug withdrawal	d
43242008	Drug withdrawal headache	d
425288007	Drugs used to treat addiction	d
228354000	Drunk driving	a
212670004	Ecstasy poisoning	d
191877009	Ecstasy type drug dependence	d
710884008	Education about managing withdrawal symptoms	d
405069002	Effect of substance addiction	d
191805002	Episodic acute alcoholic intoxication in alcoholism	a
191812006	Episodic chronic alcoholism	a
191820008	Episodic opioid dependence	d
16076691000119100	Episodic polysubstance dependence	d
28000008	Erythroxylum coca	d
442669008	Ethanol in blood specimen above legal threshold for operating vehicle	a
709460009	Exposure to alcohol	a
781698003	Exposure to methamphetamine	d
711118008	Facilitation of alcohol abuse recovery	a
391102000	Failed heroin detoxification	d
102897001	Feeling intoxicated	a
228364009	Feels afraid of being an alcoholic	a
228358002	Feels effect of alcohol at work	a
426001001	Fentanyl dependence	d
295193008	Fentanyl overdose	d
295196000	Fentanyl overdose of undetermined intent	d
290201006	Fentanyl poisoning	d

SNOMED-CT-AU code	Description	Whether used to identify problematic drug use (d), alcohol use (a) or both (b)
290204003	Fentanyl poisoning of undetermined intent	d
274776000	Finding of alcohol in blood	a
274777009	Finding of cocaine in blood	d
274778004	Finding of hallucinogen in blood	d
228273003	Finding relating to alcohol drinking behavior	a
228366006	Finding relating to drug misuse behavior	d
191853003	Glue sniffing dependence	d
191857002	Glue sniffing dependence in remission	d
191855005	Glue sniffing dependence, continuous	d
191856006	Glue sniffing dependence, episodic	d
161466001	H/O: alcoholism	a
255654008	Hallucinogen	d
74851005	Hallucinogen abuse	d
50933003	Hallucinogen delusional disorder	d
38247002	Hallucinogen dependence	d
191851001	Hallucinogen dependence in remission	d
724695007	Hallucinogen dependence with current use	d
191849000	Hallucinogen dependence, continuous	d
191850000	Hallucinogen dependence, episodic	d
32009006	Hallucinogen hallucinosis	d
121341003	Hallucinogen identification	d
50320000	Hallucinogen intoxication	d
40571009	Hallucinogen intoxication delirium	d
1383008	Hallucinogen mood disorder	d
15277004	Hallucinogen-induced anxiety disorder	d
53050002	Hallucinogen-induced organic mental disorder	d
373469002	Hallucinogenic agent	d
229006007	Hallucinogenic mushrooms	d
32553006	Hangover	a
724703003	Harmful pattern of use of substituted amphetamine	d
773000004	Harmful use of anxiolytic	d
724712001	Harmful use of dissociative drug	d
772999000	Harmful use of hypnotic	d
724713006	Harmful use of ketamine	d
230483008	Headache associated with substance abuse or withdrawal	d
231477003	Heroin dependence	d
295174006	Heroin overdose	d
295176008	Heroin overdose of undetermined intent	d
290183003	Heroin poisoning of undetermined intent	d
442766007	High alcohol level in blood	a
441685000	High ethanol level in blood	a
371434005	History of alcohol abuse	a
288761000119104	History of amphetamine abuse	d

© 2024 Spike E et al. *JAMA Network Open*

SNOMED-CT-AU code	Description	Whether used to identify problematic drug use (d), alcohol use (a) or both (b)
288771000119105	History of anxiolytic abuse	d
288781000119108	History of cannabis abuse	d
288791000119106	History of cocaine abuse	d
288801000119107	History of hallucinogen abuse	d
288811000119105	History of heroin abuse	d
699010009	History of inhalant intoxication	d
27660001000004100	History of methamphetamine abuse	d
715200006	History of novel psychoactive substance misuse	d
12275951000119100	History of opiate therapy	d
288841000119109	History of sedative hypnotic abuse	d
135321000119107	Hypersomnia caused by alcohol	a
724656006	Hypnotic dependence	d
724658007	Hypnotic dependence with current use	d
361149008	Hypnotic intoxication	d
231461004	Hypnotic or anxiolytic abuse	d
268640002	Hypnotic or anxiolytic dependence	d
191827006	Hypnotic or anxiolytic dependence in remission	d
191825003	Hypnotic or anxiolytic dependence, continuous	d
191826002	Hypnotic or anxiolytic dependence, episodic	d
724666003	Hypnotic substance withdrawal with perceptual disturbances	d
724660009	Hypnotic withdrawal	d
724663006	Hypnotic withdrawal without complication	d
313915006	Hypnotic-induced organic mental disorder	d
21000000	Idiosyncratic intoxication	a
5002000	Inhalant dependence	d
86391000119101	Inhalant dependence, continuous	d
86401000119104	Inhalant dependence, episodic	d
60901005	Inhalant intoxication	d
18689007	Inhalant intoxication delirium	d
135311000119100	Insomnia caused by alcohol	a
296293008	Intentional amphetamine overdose	d
291241005	Intentional amphetamine poisoning	d
242832005	Intentional benzodiazepine overdose	d
295168006	Intentional buprenorphine overdose	d
290176005	Intentional buprenorphine poisoning	d
296303002	Intentional cannabis overdose	d
291247009	Intentional cannabis poisoning	d
290544006	Intentional cocaine poisoning	d
242829007	Intentional diamorphine overdose	d
295195001	Intentional fentanyl overdose	d
290203009	Intentional fentanyl poisoning	d
290182008	Intentional heroin poisoning	d
295510002	Intentional ketamine overdose	d

SNOMED-CT-AU code	Description	Whether used to identify problematic drug use (d), alcohol use (a) or both (b)
290492008	Intentional ketamine poisoning	d
295568001	Intentional local cocaine overdose	d
295186009	Intentional morphine overdose	d
290193005	Intentional morphine poisoning	d
290221007	Intentional narcotic poisoning	d
242828004	Intentional opiate analgesic overdose	d
296323001	Intentional overdose by cocaine	d
296327000	Intentional overdose by crack cocaine	d
296283001	Intentional overdose of non-barbiturate hypnotic	d
291263001	Intentional poisoning by crack cocaine	d
461171000124100	Intentional poisoning by ethyl alcohol	a
461061000124108	Intentional psychostimulant overdose	d
724710009	Intoxication caused by dissociative drug	d
724711008	Intoxication caused by ketamine	d
772808000	Intoxication caused by recreational drug misuse	d
145101000119102	Intravenous cocaine abuse	d
295507009	Ketamine overdose	d
295511003	Ketamine overdose of undetermined intent	d
290493003	Ketamine poisoning of undetermined intent	d
55571001	Ketoacidosis due to acute alcohol intoxication	a
69482004	Korsakoff's psychosis	a
191471000	Korsakov's alcoholic psychosis with peripheral neuritis	a
247703006	LSD reaction	d
231475006	Librium dependence	d
295566002	Local cocaine overdose	d
295570005	Local cocaine overdose of undetermined intent	d
347841000119106	Long-term current use of cannabis	d
15698006	Lysergic acid diethylamide	d
231468005	Lysergic acid diethylamide dependence	d
440671000124106	Management of alcohol intake	a
710059009	Management of withdrawal symptom	d
733460004	Marijuana user	d
390821000	Mental health addiction programs	d
391211007	Mental health addiction programs - 1-2 contacts/week	d
391212000	Mental health addiction programs - 1-3 contacts/month	d
391206005	Mental health addiction programs - 24 hour intensive	d
391205009	Mental health addiction programs - 24 hour not intensive	d
391210008	Mental health addiction programs - 3-5 contacts/week	d
391213005	Mental health addiction programs - <1 contact/month	d
391209003	Mental health addiction programs - daily intensive	d

© 2024 Spike E et al. *JAMA Network Open*

SNOMED-CT-AU code	Description	Whether used to identify problematic drug use (d), alcohol use (a) or both (b)
391207001	Mental health addiction programs - full day : day care	d
391208006	Mental health addiction programs - part day : day care	d
231469002	Mescaline dependence	d
231478008	Methadone dependence	d
429512006	Methadone misuse	d
699449003	Methamphetamine abuse	d
426873000	Methamphetamine dependence	d
12398571000119100	Methamphetamine intoxication	d
304605000	Methanol abuse	a
212809004	Methyl alcohol causing toxic effect	a
288459003	Methylenedioxymethamphetamine	d
724704009	Methylenedioxymethamphetamine dependence with current use	d
713583005	Mild alcohol dependence	a
191939002	Misuse of prescription only drugs	d
44870007	Misused drugs in past	d
105550004	Misused volatile solvents in past	d
714829008	Moderate alcohol dependence	a
710986006	Monitoring drug withdrawal	d
724721000	Mood disorder caused by ketamine	d
724707002	Mood disorder caused by methylenedioxymethamphetamine	d
762328006	Mood disorder caused by stimulant	d
723928009	Mood disorder with depressive symptoms caused by alcohol	a
724678004	Mood disorder with depressive symptoms caused by anxiolytic	d
724690002	Mood disorder with depressive symptoms caused by cocaine	d
762336002	Mood disorder with depressive symptoms caused by hallucinogen	d
724677009	Mood disorder with depressive symptoms caused by hypnotic	d
724676000	Mood disorder with depressive symptoms caused by sedative	d
762329003	Mood disorder with depressive symptoms caused by stimulant	d
723929001	Mood disorder with manic symptoms caused by alcohol	a
724681009	Mood disorder with manic symptoms caused by anxiolytic	d
724691003	Mood disorder with manic symptoms caused by cocaine	d
762335003	Mood disorder with manic symptoms caused by hallucinogen	d
724680005	Mood disorder with manic symptoms caused by hypnotic	d
724679007	Mood disorder with manic symptoms caused by sedative	d

SNOMED-CT-AU code	Description	Whether used to identify problematic drug use (d), alcohol use (a) or both (b)
724684001	Mood disorder with mixed depressive and manic symptoms caused by anxiolytic	d
724692005	Mood disorder with mixed depressive and manic symptoms caused by cocaine	d
762337006	Mood disorder with mixed depressive and manic symptoms caused by hallucinogen	d
724683007	Mood disorder with mixed depressive and manic symptoms caused by hypnotic	d
724682002	Mood disorder with mixed depressive and manic symptoms caused by sedative	d
762330008	Mood disorder with mixed depressive and manic symptoms caused by stimulant	d
723930006	Mood disorder with mixed manic and depressive symptoms caused by alcohol	a
231479000	Morphine dependence	d
295184007	Morphine overdose	d
295187000	Morphine overdose of undetermined intent	d
290194004	Morphine poisoning of undetermined intent	d
70545002	Narcotic drug user	d
117644007	Narcotic identification	d
290222000	Narcotic poisoning of undetermined intent	d
230443000	Narcotic withdrawal epilepsy	d
8061000175108	Narcotics anonymous	d
268645007	Nondependent alcohol abuse	a
191884001	Nondependent alcohol abuse in remission	a
191882002	Nondependent alcohol abuse, continuous	a
191883007	Nondependent alcohol abuse, episodic	a
34111000119108	Nondependent amphetamine abuse	d
268648009	Nondependent amphetamine or other psychostimulant abuse	d
414874007	Nondependent amphetamine or psychostimulant abuse in remission	d
191924003	Nondependent amphetamine or psychostimulant abuse, continuous	d
191925002	Nondependent amphetamine or psychostimulant abuse, episodic	d
191891003	Nondependent cannabis abuse	d
191895007	Nondependent cannabis abuse in remission	d
191893000	Nondependent cannabis abuse, continuous	d
191894006	Nondependent cannabis abuse, episodic	d
191916008	Nondependent cocaine abuse	d
191920007	Nondependent cocaine abuse in remission	d
191918009	Nondependent cocaine abuse, continuous	d
191919001	Nondependent cocaine abuse, episodic	d
268646008	Nondependent hallucinogen abuse	d
191901005	Nondependent hallucinogen abuse in remission	d
191899001	Nondependent hallucinogen abuse, continuous	d
191900006	Nondependent hallucinogen abuse, episodic	d
268647004	Nondependent hypnotic or anxiolytic abuse	d

© 2024 Spike E et al. *JAMA Network Open*

SNOMED-CT-AU code	Description	Whether used to identify problematic drug use (d), alcohol use (a) or both (b)
191907009	Nondependent hypnotic or anxiolytic abuse in remission	d
191905001	Nondependent hypnotic or anxiolytic abuse, continuous	d
191906000	Nondependent hypnotic or anxiolytic abuse, episodic	d
144981000119109	Nondependent intraveous amphetamine abuse	d
228374007	Notified addict	d
713775002	Novel psychoactive substance misuse	d
416119007	O/E - signs of drug withdrawal	d
762332000	Obsessive compulsive disorder caused by caused by stimulant	d
724693000	Obsessive compulsive disorder caused by cocaine	d
428819003	Opiate misuse	d
75544000	Opioid dependence	d
191821007	Opioid dependence in remission	d
724653003	Opioid dependence with current use	d
1081000119105	Opioid dependence, on agonist therapy	d
441970008	Opioid in blood specimen positive	d
77721001	Opioid intoxication	d
52866005	Opioid intoxication delirium	d
87132004	Opioid withdrawal	d
769176003	Opioid withdrawal electrical stimulator	d
288861000119108	Opioid-induced mood disorder due to opioid dependence	d
231480002	Opium dependence	d
296129007	Overdose of benzodiazepine of undetermined intent	d
296321004	Overdose of cocaine	d
296324007	Overdose of cocaine of undetermined intent	d
296325008	Overdose of crack cocaine	d
296328005	Overdose of crack cocaine of undetermined intent	d
296300004	Overdose of hallucinogenic drug	d
296305009	Overdose of lysergic acid	d
296284007	Overdose of non-barbiturate hypnotic of undetermined intent	d
242253008	Overdose of opiate	d
295213004	Overdose of opiate analgesic of undetermined intent	d
31715000	PCP delirium	d
58727001	PCP dependence	d
20871009	PCP intoxication	d
135301000119103	Parasomnia caused by alcohol	a
191477001	Pathological alcohol intoxication	a
25508008	Pathological drug intoxication	d
288041000119101	Perceptual disturbance due to alcohol withdrawal	a
723926008	Perceptual disturbances and seizures co-occurrent and due to alcohol withdrawal	a

SNOMED-CT-AU code	Description	Whether used to identify problematic drug use (d), alcohol use (a) or both (b)
724672003	Perceptual disturbances and seizures co-occurrent and due to hypnotic withdrawal	d
724728006	Perceptual disturbances and seizures co-occurrent and due to psychoactive substance withdrawal	d
724671005	Perceptual disturbances and seizures co-occurrent and due to sedative withdrawal	d
724726005	Perceptual disturbances co-occurrent and due to psychoactive substance withdrawal	d
284591009	Persistent alcohol abuse	a
417471002	Persistent substance misuse	d
425841004	Phencyclidine dependence in remission	d
41784005	Physical addiction	d
228351008	Physical tolerance to alcohol	a
22121000087104	Physical tolerance to opiate drug	d
16804008	Plant producing hallucinogen	d
45775001	Poisoning by amphetamine	d
81914009	Poisoning by benzodiazepine-based tranquilizer	d
15233006	Poisoning by cannabis derivative	d
61803000	Poisoning by central nervous system stimulant	d
9982009	Poisoning by cocaine	d
241761001	Poisoning by crack cocaine	d
291264007	Poisoning by crack cocaine of undetermined intent	d
13187008	Poisoning by heroin	d
10650000	Poisoning by ketamine	d
67893003	Poisoning by lysergide	d
60199004	Poisoning by methadone	d
111763008	Poisoning by mixed sedative	d
47836003	Poisoning by morphine	d
11196001	Poisoning by opiate AND/OR related narcotic	d
241749009	Poisoning by opiate analgesic drug	d
74264003	Poisoning by opium alkaloid	d
85975005	Poisoning by psychodysleptic	d
20260003	Poisoning by psychostimulant	d
85337000	Poisoning by sedative AND/OR hypnotic	d
445273005	Polysubstance abuse	d
51339003	Polysubstance dependence	d
237228001	Pregnancy and drug dependence	d
416437003	Preoccupied with substance misuse	d
228281002	Problem drinker	a
416246006	Prolonged high dose use of cannabis	d
299995007	Propan-1-ol	a
91388009	Psychoactive substance abuse	d
2403008	Psychoactive substance dependence	d
724724008	Psychoactive substance dependence with current use	d

© 2024 Spike E et al. *JAMA Network Open*

SNOMED-CT-AU code	Description	Whether used to identify problematic drug use (d), alcohol use (a) or both (b)
11061003	Psychoactive substance use disorder	d
724725009	Psychoactive substance withdrawal without complication	d
396344000	Psychoactive substance-induced organic intoxication	d
74934004	Psychoactive substance-induced withdrawal syndrome	d
85758008	Psychological addiction	d
228352001	Psychological tolerance to alcohol	a
275471001	Psychostimulant dependence	d
296317006	Psychostimulant overdose	d
428370001	Psychostimulant withdrawal	d
723927004	Psychotic disorder caused by alcohol with schizophreniform symptoms	a
724675001	Psychotic disorder caused by anxiolytic	d
723936000	Psychotic disorder caused by cannabis	d
724689006	Psychotic disorder caused by cocaine	d
724696008	Psychotic disorder caused by hallucinogen	d
724674002	Psychotic disorder caused by hypnotic	d
724719005	Psychotic disorder caused by ketamine	d
15921731000119100	Psychotic disorder caused by methamphetamine	d
724706006	Psychotic disorder caused by methylenedioxymethamphetamine	d
724673008	Psychotic disorder caused by sedative	d
762325009	Psychotic disorder caused by stimulant	d
737340007	Psychotic disorder caused by synthetic cannabinoid	d
7761000119106	Psychotic disorder due to amphetamine use	d
762327001	Psychotic disorder with delusions caused by stimulant	d
762326005	Psychotic disorder with hallucinations caused by stimulant	d
735750005	Psychotic disorder with schizophreniform symptoms caused by cocaine	d
772133000	Recreational drug misuse withdrawal	d
416751004	Reduced drugs misuse	d
38670004	Referral to alcoholism rehabilitation service	a
390857005	Referral to community alcohol team	a
417096006	Referral to community drug and alcohol team	d
4266003	Referral to drug addiction rehabilitation service	d
431260004	Referral to specialist alcohol treatment service	a
442444001	Referral to specialist alcohol treatment service declined by patient	a
228353006	Reverse tolerance to alcohol	a
405137009	Risk control behavior: alcohol use	a
64386003	Sedative abuse	d
302507002	Sedative amnestic disorder	d
427327003	Sedative dependence	d

SNOMED-CT-AU code	Description	Whether used to identify problematic drug use (d), alcohol use (a) or both (b)
724657002	Sedative dependence with current use	d
296015009	Sedative overdose	d
724665004	Sedative substance withdrawal with perceptual disturbances	d
23601000119102	Sedative withdrawal	d
361151007	Sedative withdrawal delirium	d
724668002	Sedative withdrawal with seizure	d
724662001	Sedative withdrawal without complication	d
5444000	Sedative, hypnotic AND/OR anxiolytic intoxication delirium	d
361150008	Sedative, hypnotic AND/OR anxiolytic withdrawal delirium	d
1686006	Sedative, hypnotic AND/OR anxiolytic-induced anxiety disorder	d
28864000	Sedative, hypnotic AND/OR anxiolytic-induced mood disorder	d
301643003	Sedative, hypnotic AND/OR anxiolytic-induced persisting amnestic disorder	d
59651006	Sedative, hypnotic AND/OR anxiolytic-induced persisting dementia	d
1973000	Sedative, hypnotic AND/OR anxiolytic-induced psychotic disorder with delusions	d
47664006	Sedative, hypnotic AND/OR anxiolytic-induced psychotic disorder with hallucinations	d
12380008	Sedative, hypnotic AND/OR anxiolytic-induced sexual dysfunction	d
57588009	Sedative, hypnotic AND/OR anxiolytic-induced sleep disorder	d
312098001	Sedative, hypnotic AND/OR anxiolytic-related disorder	d
700187009	Seen in drug misuse clinic	d
724670006	Seizure co-occurrent and due to anxiolytic withdrawal	d
735235000	Seizure co-occurrent and due to drug withdrawal	d
724669005	Seizure co-occurrent and due to hypnotic withdrawal	d
724727001	Seizure co-occurrent and due to psychoactive substance withdrawal	d
713862009	Severe alcohol dependence	a
762333005	Sexual dysfunction caused by stimulant	d
724709004	Sexual dysfunction caused by substituted amphetamine drug	d
723937009	Sleep disorder caused by cannabis	d
12398651000119100	Sleep disorder caused by methamphetamine	d
762334004	Sleep disorder caused by stimulant	d
737342004	Sleep disorder caused by synthetic cannabinoid	d
428495004	Solvent misuse	d
441527004	Stimulant abuse	d
442406005	Stimulant dependence	d
762323002	Stimulant intoxication	d
415658005	Substance misuse behavior	d

© 2024 Spike E et al. *JAMA Network Open*

SNOMED-CT-AU code	Description	Whether used to identify problematic drug use (d), alcohol use (a) or both (b)
415659002	Substance misuse clinical management plan agreed	d
415660007	Substance misuse decreased	d
415661006	Substance misuse increased	d
415662004	Substance misuse monitoring	d
415663009	Substance misuse monitoring status	d
273850003	Substance use disorder diagnostic schedule	d
386449006	Substance use treatment: alcohol withdrawal	a
386450006	Substance use treatment: drug withdrawal	d
461181000124102	Suicide attempt by alcohol poisoning	a
461031000124104	Suicide attempt by benzodiazepine overdose	d
461291000124108	Suicide attempt by buprenorphine and naloxone overdose	d
461091000124100	Suicide attempt by cocaine overdose	d
461011000124105	Suicide attempt by fentanyl overdose	d
461001000124107	Suicide attempt by heroin overdose	d
461051000124106	Suicide attempt by psychostimulant overdose	d
415685003	Suspected alcohol abuse	a
737336003	Synthetic cannabinoid abuse	d
737337007	Synthetic cannabinoid dependence	d
737338002	Synthetic cannabinoid withdrawal	d
762505006	Synthetic cathinone dependence	d
762671008	Synthetic cathinone intoxication	d
762672001	Synthetic cathinone withdrawal	d
95918004	Therapeutic drug dependence	d
228411009	Time since stopped drug misuse	d
228349009	Tolerance to alcohol	a
365978002	Tolerance to alcohol - finding	a
67426006	Toxic effect of alcohol	a
87460008	Toxic effect of amyl alcohol	a
4953006	Toxic effect of butyl alcohol	a
89507002	Toxic effect of denatured alcohol	a
82782008	Toxic effect of ethyl alcohol	a
57346004	Toxic effect of fusel oil	a
212813006	Toxic effect of isopropyl alcohol	a
6749002	Toxic effect of propyl alcohol	a
722971006	Tremor due to drug withdrawal	d
228344004	Unable to stop drinking before intoxication	a
85561006	Uncomplicated alcohol withdrawal	a
10028000	Uncomplicated sedative, hypnotic AND/OR anxiolytic withdrawal	d
135827004	Under care of community alcohol team	a
744857009	Under care of drug misuse service	d
10939881000119100	Unhealthy alcohol drinking behavior	a
724700000	Volatile inhalant dependence with current use	d

© 2024 Spike E et al. *JAMA Network Open*

SNOMED-CT-AU code	Description	Whether used to identify problematic drug use (d), alcohol use (a) or both (b)
724701001	Volatile inhalant withdrawal	d
719340007	Withdrawn from alcohol detoxification program	a

© 2024 Spike E et al. *JAMA Network Open*

eAppendix 6. Further Detail Regarding Covariate and Model Selection

Covariate selection

Selection of variables for inclusion in our statistical models as covariates was based on established or strongly suspected influences of the variable on both criminal legal system sanction types and mortality, i.e. satisfying the definition of a classical confounder, and was guided by the below Directed Acyclic Graph (DAG). Further explanation regarding some variables in the DAG and how they were measured in our study are indicated by asterisks in the DAG, with explanations following the DAG.

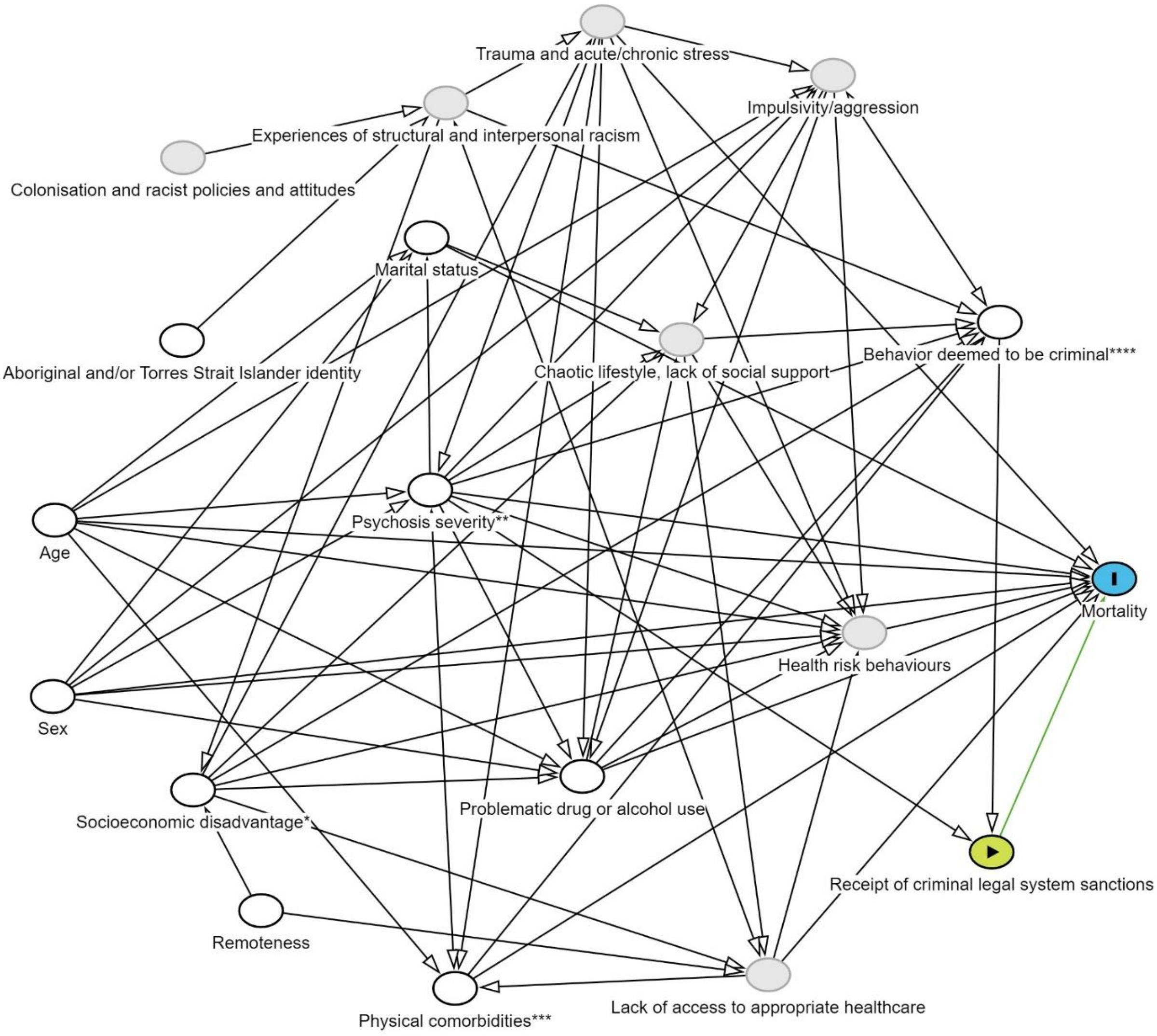

Legend:

Measured covariate **Unmeasured variable** **Exposure** **Outcome**

Green arrow indicates causal path of interest. DAG produced using dagitty.com.

*Socioeconomic disadvantage, which encompasses both individual and area-level socioeconomic disadvantage, was measured using residential area-level Index of Relative Socioeconomic Disadvantage[6] as a proxy.

**Psychosis severity was measured using involuntary index admission as a proxy.

***Physical comorbidities were measured using the updated Charlson Comorbidity Index[7] score.

****'Behaviour deemed to be criminal' was measured using offense history at study entry (categorized as violent, non-violent only, or none). We recognize that being charged with and/or convicted of an offense is a reflection not only of individual behaviors (which, particularly in the case of violent behaviors, often imply a degree of aggression or impulsivity), but also a variety of other individual, societal, and systemic factors. These include the wider legal system and which behaviors are deemed criminal (and by whom, and in what context), as well as law enforcement activity.

Aboriginal and/or Torres Strait Islander identity was included as a covariate given that it is known to be associated with both the exposure (criminal legal system sanction type)[8] and outcome (mortality).[9] However, as depicted in the above DAG, we believe that these associations are mediated by the impacts of colonization and

racism. We also acknowledge that Aboriginal and/or Torres Strait Islander identity was recorded in the administrative data collections used in our study based on self-report at the time of administrative data collection, and that the response options were defined by the organization collecting the data rather than by the individual patient/participant. Furthermore, our decision to categorize this variable as 'Aboriginal and/or Torres Strait Islander identity' rather than distinguishing between Aboriginal identity, Torres Strait Islander identity, or both, was based on the fact that some of the source datasets in our study did not make this distinction and only included a single category indicating Aboriginal and/or Torres Strait Islander identity. We ascertained Aboriginal and/or Torres Strait Islander identity from all source datasets in which this was recorded (APDC, EDDC, RoD, OIMS and COD-URF) by applying a validated multi-stage median algorithm within and across datasets; this algorithm has been shown to have sensitivities of 90.7% and 87.7% and specificities of 99.3% and 99.4% when applied to the NSW APDC and EDDC respectively.[10]

Model selection

Model selection was theory-driven based on prior knowledge and was specified *a priori*, as is an accepted practice in epidemiological studies.[11] We took a progressive approach to confounder adjustment to allow the impacts of sociodemographic factors alone on the effect estimates to be distinguished from the impacts of a comprehensive range of factors including sociodemographic factors, health-related factors and baseline offense history. Model 1 adjusted for age and sex only, as these are major contributors to the risks of both criminal legal system involvement and mortality at any given time, and are taken into account in almost all mortality reporting either via adjustment, standardization or stratification. Model 2 further adjusted for an expanded range of sociodemographic characteristics, i.e. socioeconomic status, remoteness, Aboriginal and/or Torres Strait Islander identity and marital status. Model 3, which was our maximally adjusted model, further adjusted for all relevant covariates that were identified as potential confounding variables and were available to measure in our source data collections, i.e. health-related factors (problematic drug use, problematic alcohol use, psychosis severity, and physical comorbidities) and baseline offense history.

eTable 1. Participant Characteristics by Recent (Past 2 Years) Criminal Sanction Type at Study Entry for Participants Aged <65 Years at Entry

	No sanction (n=64,065)		Community sanction (n=5,940)		Diversion (n=1,028)		Current imprisonment (n=1,132)		Prior imprisonment (n=2,676)		Total (n=74,841)	
	**n**	**(%)**	**n**	**(%)**	**n**	**(%)**	**n**	**(%)**	**n**	**(%)**	**n**	**(%)**
**Sex**												
Female or other[a]	28,400	(44.3)	1,417	(23.9)	218	(21.2)	117	(10.3)	402	(15.0)	30,554	(40.8)
**Age group**												
18-24 years	14,102	(22.0)	1,657	(27.9)	239	(23.2)	281	(24.8)	636	(23.8)	16,915	(22.6)
25-34 years	17,118	(26.7)	2,096	(35.3)	354	(34.4)	474	(41.9)	1,166	(43.6)	21,208	(28.3)
35-44 years	14,979	(23.4)	1,479	(24.9)	267	(26.0)	259	(22.9)	660	(24.7)	17,644	(23.6)
45-54 years	10,982	(17.1)	571	(9.6)	122	(11.9)	91	(8.0)	187	(7.0)	11,953	(16.0)
55-64 years	6,884	(10.7)	137	(2.3)	46	(4.5)	27	(2.4)	27	(1.0)	7,121	(9.5)
**Aboriginal and/or Torres Strait Islander identity**												
Yes	5,243	(8.2)	1,225	(20.6)	174	(16.9)	356	(31.4)	1,809	(67.6)	7,865	(10.5)
No	58,578	(91.4)	4,715	(79.4)	854	(83.1)	776	(68.6)	867	(32.4)	66,732	(89.2)
Missing or unknown	244	(0.4)	0	(0.0)	0	(0.0)	0	(0.0)	0	(0.0)	244	(0.3)
**Residential Index of Relative Socioeconomic Disadvantage**												

© 2024 Spike E et al. *JAMA Network Open*

	No sanction (n=64,065)		Community sanction (n=5,940)		Diversion (n=1,028)		Current imprisonment (n=1,132)		Prior imprisonment (n=2,676)		Total (n=74,841)	
	n	(%)	n	(%)	n	(%)	n	(%)	n	(%)	n	(%)
Least disadvantaged	24,816	(38.7)	1,797	(30.3)	384	(37.4)	266	(23.5)	680	(25.4)	27,943	(37.3)
Moderately disadvantaged	19,572	(30.6)	1,947	(32.8)	284	(27.6)	117	(10.3)	901	(33.7)	22,821	(30.5)
Most disadvantaged	16,000	(25.0)	1,821	(30.7)	280	(27.2)	735	(64.9)	909	(34.0)	19,745	(26.4)
Interstate resident	2,074	(3.2)	75	(1.3)	22	(2.1)	6	(0.5)	16	(0.6)	2,193	(2.9)
Missing or unknown	1,603	(2.5)	300	(5.1)	58	(5.6)	8	(0.7)	170	(6.4)	2,139	(2.9)
**Residential remoteness**												
Major cities	45,164	(70.5)	3,828	(64.4)	769	(74.8)	1,001	(88.4)	1,738	(64.9)	52,500	(70.1)
Inner regional	11,903	(18.6)	1,272	(21.4)	147	(14.3)	100	(8.8)	551	(20.6)	13,973	(18.7)
Outer regional/remote	3,321	(5.2)	465	(7.8)	32	(3.1)	17	(1.5)	201	(7.5)	4,036	(5.4)
Interstate resident	2,074	(3.2)	75	(1.3)	22	(2.1)	6	(0.5)	16	(0.6)	2,193	(2.9)
Missing or unknown	1,603	(2.5)	300	(5.1)	58	(5.6)	8	(0.7)	170	(6.4)	2,139	(2.9)
**Married (including de facto)**												
Yes	13,821	(21.6)	685	(11.5)	123	(12.0)	211	(18.6)	302	(11.3)	15,142	(20.2)
No	44,885	(70.1)	4,771	(80.3)	797	(77.5)	827	(73.1)	2,151	(80.4)	53,431	(71.4)
Missing or unknown	5,359	(8.4)	484	(8.1)	108	(10.5)	94	(8.3)	223	(8.3)	6,268	(8.4)
**Problematic drug use**												

© 2024 Spike E et al. *JAMA Network Open*

	No sanction (n=64,065)		Community sanction (n=5,940)		Diversion (n=1,028)		Current imprisonment (n=1,132)		Prior imprisonment (n=2,676)		Total (n=74,841)	
	**n**	**(%)**	**n**	**(%)**	**n**	**(%)**	**n**	**(%)**	**n**	**(%)**	**n**	**(%)**
Yes	21,805	(34.0)	4,301	(72.4)	557	(54.2)	651	(57.5)	2,219	(82.9)	29,533	(39.5)
**Problematic alcohol use**												
Yes	11,133	(17.4)	2,263	(38.1)	312	(30.4)	307	(27.1)	1,154	(43.1)	15,169	(20.3)
**Involuntary index admission**												
Yes	30,817	(48.1)	3,049	(51.3)	670	(65.2)	230	(20.3)	1,322	(49.4)	36,088	(48.2)
**Charlson Comorbidity Index (past year of age)**												
0	62,572	(97.7)	5,735	(96.5)	996	(96.9)	1,091	(96.4)	2,554	(95.4)	72,948	(97.5)
1	696	(1.1)	58	(1.0)	17	(1.7)	18	(1.6)	23	(0.9)	812	(1.1)
≥2	797	(1.2)	147	(2.5)	15	(1.5)	23	(2.0)	99	(3.7)	1,081	(1.4)
**Offense history**												
None	53,647	(83.7)	0	(0.0)	0	(0.0)	39	(3.4)	77	(2.9)	53,763	(71.8)
Non-violent offenses only	4,660	(7.3)	2,400	(40.4)	266	(25.9)	134	(11.8)	401	(15.0)	7,861	(10.5)
Violent offenses	5,741	(9.0)	3,540	(59.6)	762	(74.1)	959	(84.7)	2,198	(82.1)	13,200	(17.6)
Unknown offenses only	17	(0.0)	0	(0.0)	0	(0.0)	0	(0.0)	0	(0.0)	17	(0.0)
**Died**												
Yes	5,887	(9.2)	525	(8.8)	115	(11.2)	91	(8.0)	265	(9.9)	6,883	(9.2)

[a]Other category not reported separately due to having less than 5 participants in the entire cohort; other included intersex.

© 2024 Spike E et al. *JAMA Network Open*

eTable 2. Participant Characteristics by Recent (Past 2 Years) Criminal Sanction Type at Study Entry for Participants Aged ≥65 Years at Entry

	No sanction (n=8,171)		Community sanction (n=38)		Diversion (n=15)		Current imprisonment (n<5)		Prior imprisonment (n<5)		Total (n=8,230)	
	**n**	**(%)**	**n**	**(%)**	**n**	**(%)**	**n**	**(%)**	**n**	**(%)**	**n**	**(%)**
**Sex**												
Female or other[a]	5,223	(63.9)	10	(26.3)	<5	NS	0	(0.0)	0	(0.0)	5,237	(63.6)
**Age group**												
≥65 years	8,171	(100.0)	38	(100.0)	15	(100.0)	<5	(100.0)	<5	(100.0)	8,230	(100.0)
**Aboriginal and/or Torres Strait Islander identity**												
Yes	117	(1.4)	<5	NS	<5	NS	0	(0.0)	0	(0.0)	119	(1.4)
No	8,044	(98.4)	37	(97.4)	14	(93.3)	<5	NS	<5	NS	8,101	(98.4)
Missing or unknown	10	(0.1)	0	(0.0)	0	(0.0)	0	(0.0)	0	(0.0)	10	(0.1)
**Residential Index of Relative Socioeconomic Disadvantage**												
Least disadvantaged	3,583	(43.9)	16	(42.1)	6	(40.0)	<5	NS	0	(0.0)	3,609	(43.9)
Moderately disadvantaged	2,573	(31.5)	15	(39.5)	5	(33.3)	0	(0.0)	<5	NS	2,594	(31.5)
Most disadvantaged	1,877	(23.0)	<5	NS	<5	NS	0	(0.0)	<5	NS	1,885	(22.9)
Interstate resident	90	(1.1)	0	(0.0)	0	(0.0)	0	(0.0)	0	(0.0)	90	(1.1)
Missing or unknown	48	(0.6)	<5	NS	<5	NS	0	(0.0)	0	(0.0)	52	(0.6)

© 2024 Spike E et al. *JAMA Network Open*

	No sanction (n=8,171)		Community sanction (n=38)		Diversion (n=15)		Current imprisonment (n<5)		Prior imprisonment (n<5)		Total (n=8,230)	
	**n**	**(%)**	**n**	**(%)**	**n**	**(%)**	**n**	**(%)**	**n**	**(%)**	**n**	**(%)**
**Residential remoteness**												
Major cities	6,009	(73.5)	26	(68.4)	12	(80.0)	<5	NS	<5	NS	6,052	(73.5)
Inner regional	1,587	(19.4)	7	(18.4)	<5	NS	0	(0.0)	0	(0.0)	1,596	(19.4)
Outer regional/remote	437	(5.3)	<5	NS	0	(0.0)	0	(0.0)	<5	NS	440	(5.3)
Interstate resident	90	(1.1)	0	(0.0)	0	(0.0)	0	(0.0)	0	(0.0)	90	(1.1)
Missing or unknown	48	(0.6)	<5	NS	<5	NS	0	(0.0)	0	(0.0)	52	(0.6)
**Married (including de facto)**												
Yes	2,837	(34.7)	12	(31.6)	<5	NS	<5	NS	0	(0.0)	2,854	(34.7)
No	4,841	(59.2)	21	(55.3)	8	(53.3)	<5	NS	<5	NS	4,874	(59.2)
Missing or unknown	493	(6.0)	5	(13.2)	<5	NS	0	(0.0)	<5	NS	502	(6.1)
**Problematic drug use**												
Yes	411	(5.0)	<5	NS	0	(0.0)	<5	NS	0	(0.0)	415	(5.0)
**Problematic alcohol use**												
Yes	537	(6.6)	14	(36.8)	<5	NS	0	(0.0)	<5	NS	555	(6.7)
**Involuntary index admission**												
Yes	2,571	(31.5)	22	(57.9)	11	(73.3)	<5	NS	<5	NS	2,607	(31.7)

© 2024 Spike E et al. *JAMA Network Open*

	No sanction (n=8,171)		Community sanction (n=38)		Diversion (n=15)		Current imprisonment (n<5)		Prior imprisonment (n<5)		Total (n=8,230)	
	**n**	**(%)**	**n**	**(%)**	**n**	**(%)**	**n**	**(%)**	**n**	**(%)**	**n**	**(%)**
**Charlson Comorbidity Index (past year of age)**												
0	7,074	(86.6)	32	(84.2)	12	(80.0)	<5	NS	<5	NS	7,123	(86.5)
1	568	(7.0)	<5	NS	<5	NS	<5	NS	0	(0.0)	572	(7.0)
≥2	529	(6.5)	<5	NS	<5	NS	0	(0.0)	0	(0.0)	535	(6.5)
**Offense history**												
None	8,021	(98.2)	0	(0.0)	0	(0.0)	<5	NS	0	(0.0)	8,022	(97.5)
Non-violent offenses only	65	(0.8)	13	(34.2)	<5	NS	0	(0.0)	<5	NS	82	(1.0)
Violent offenses	85	(1.0)	25	(65.8)	12	(80.0)	<5	NS	<5	NS	126	(1.5)
Unknown offenses only	0	(0.0)	0	(0.0)	0	(0.0)	0	(0.0)	0	(0.0)	0	(0.0)
**Died**												
Yes	4,448	(54.4)	16	(42.1)	7	(46.7)	0	(0.0)	<5	NS	4,472	(54.3)

NS=not shown due to corresponding n<5.

[a]Other category not reported separately due to having less than 5 participants in the entire cohort; other included intersex.

eTable 3. Participant Characteristics by Recent (Past 2 Years) Criminal Sanction Type at Last Observation Aged <65 Years

	No sanction (n=65,158)		Community sanction (n=4,519)		Diversion (n=1,351)		Current imprisonment (n=1,197)		Prior imprisonment (n=2,616)		Total (n=74,841)	
	**n**	**(%)**	**n**	**(%)**	**n**	**(%)**	**n**	**(%)**	**n**	**(%)**	**n**	**(%)**
**Sex**												
Female or other[a]	28,436	(43.6)	1,192	(26.4)	373	(27.6)	90	(7.5)	463	(17.7)	30,554	(40.8)
**Age group**												
18-24 years	2,354	(3.6)	341	(7.5)	128	(9.5)	88	(7.4)	184	(7.0)	3,095	(4.1)
25-34 years	10,647	(16.3)	1,190	(26.3)	378	(28.0)	462	(38.6)	881	(33.7)	13,558	(18.1)
35-44 years	16,787	(25.8)	1,547	(34.2)	399	(29.5)	425	(35.5)	948	(36.2)	20,106	(26.9)
45-54 years	16,398	(25.2)	1,041	(23.0)	310	(22.9)	186	(15.5)	481	(18.4)	18,416	(24.6)
55-64 years	18,972	(29.1)	400	(8.9)	136	(10.1)	36	(3.0)	122	(4.7)	19,666	(26.3)
**Aboriginal and/or Torres Strait Islander identity**												
Yes	5,299	(8.1)	1,015	(22.5)	262	(19.4)	431	(36.0)	858	(32.8)	7,865	(10.5)
No	59,615	(91.5)	3,504	(77.5)	1,089	(80.6)	766	(64.0)	1,758	(67.2)	66,732	(89.2)
Missing or unknown	244	(0.4)	0	(0.0)	0	(0.0)	0	(0.0)	0	(0.0)	244	(0.3)
**Residential Index of Relative Socioeconomic Disadvantage**												
Least disadvantaged	25,037	(38.4)	1,405	(31.1)	490	(36.3)	326	(27.2)	685	(26.2)	27,943	(37.3)

© 2024 Spike E et al. *JAMA Network Open*

	No sanction (n=65,158)		Community sanction (n=4,519)		Diversion (n=1,351)		Current imprisonment (n=1,197)		Prior imprisonment (n=2,616)		Total (n=74,841)	
	**n**	**(%)**	**n**	**(%)**	**n**	**(%)**	**n**	**(%)**	**n**	**(%)**	**n**	**(%)**
Moderately disadvantaged	19,730	(30.3)	1,483	(32.8)	392	(29.0)	363	(30.3)	853	(32.6)	22,821	(30.5)
Most disadvantaged	16,592	(25.5)	1,382	(30.6)	389	(28.8)	454	(37.9)	928	(35.5)	19,745	(26.4)
Interstate resident	2,072	(3.2)	67	(1.5)	19	(1.4)	8	(0.7)	27	(1.0)	2,193	(2.9)
Missing or unknown	1,727	(2.7)	182	(4.0)	61	(4.5)	46	(3.8)	123	(4.7)	2,139	(2.9)
**Residential remoteness**												
Major cities	45,966	(70.5)	2,962	(65.5)	975	(72.2)	836	(69.8)	1,761	(67.3)	52,500	(70.1)
Inner regional	11,987	(18.4)	1,008	(22.3)	235	(17.4)	222	(18.5)	521	(19.9)	13,973	(18.7)
Outer regional/remote	3,406	(5.2)	300	(6.6)	61	(4.5)	85	(7.1)	184	(7.0)	4,036	(5.4)
Interstate resident	2,072	(3.2)	67	(1.5)	19	(1.4)	8	(0.7)	27	(1.0)	2,193	(2.9)
Missing or unknown	1,727	(2.7)	182	(4.0)	61	(4.5)	46	(3.8)	123	(4.7)	2,139	(2.9)
**Married (including de facto)**												
Yes	13,981	(21.5)	564	(12.5)	143	(10.6)	145	(12.1)	309	(11.8)	15,142	(20.2)
No	45,599	(70.0)	3,658	(80.9)	1,102	(81.6)	969	(81.0)	2,103	(80.4)	53,431	(71.4)
Missing or unknown	5,578	(8.6)	297	(6.6)	106	(7.8)	83	(6.9)	204	(7.8)	6,268	(8.4)
**Problematic drug use**												
Yes	22,326	(34.3)	3,345	(74.0)	780	(57.7)	965	(80.6)	2,117	(80.9)	29,533	(39.5)

© 2024 Spike E et al. *JAMA Network Open*

	No sanction (n=65,158)		Community sanction (n=4,519)		Diversion (n=1,351)		Current imprisonment (n=1,197)		Prior imprisonment (n=2,616)		Total (n=74,841)	
	**n**	**(%)**	**n**	**(%)**	**n**	**(%)**	**n**	**(%)**	**n**	**(%)**	**n**	**(%)**
**Problematic alcohol use**												
Yes	11,830	(18.2)	1,541	(34.1)	362	(26.8)	436	(36.4)	1,000	(38.2)	15,169	(20.3)
**Involuntary index admission**												
Yes	31,175	(47.8)	2,332	(51.6)	767	(56.8)	552	(46.1)	1,262	(48.2)	36,088	(48.2)
**Charlson Comorbidity Index (past year of age)**												
0	60,700	(93.2)	3,995	(88.4)	1,155	(85.5)	1,054	(88.1)	2,248	(85.9)	69,152	(92.4)
1	1,809	(2.8)	86	(1.9)	50	(3.7)	17	(1.4)	40	(1.5)	2,002	(2.7)
≥2	2,649	(4.1)	438	(9.7)	146	(10.8)	126	(10.5)	328	(12.5)	3,687	(4.9)
**Offense conviction history**												
None	51,075	(78.4)	1,568	(34.7)	576	(42.6)	125	(10.4)	419	(16.0)	53,763	(71.8)
Non-violent offenses only	5,823	(8.9)	1,107	(24.5)	257	(19.0)	178	(14.9)	496	(19.0)	7,861	(10.5)
Violent offenses	8,246	(12.7)	1,843	(40.8)	517	(38.3)	894	(74.7)	1,700	(65.0)	13,200	(17.6)
Unknown offenses only	14	(0.0)	<5	NS	<5	NS	0	(0.0)	<5	NS	17	(0.0)
**Died**												
Yes	4,692	(7.2)	550	(12.2)	189	(14.0)	25	(2.1)	334	(12.8)	5,790	(7.7)

NS=not shown due to corresponding n<5.

[a]Other category not reported separately due to having less than 5 participants in the entire cohort; other included intersex.

© 2024 Spike E et al. *JAMA Network Open*

eTable 4. Participant Characteristics by Recent (Past 2 Years) Criminal Sanction Type at Last Observation Aged ≥65 Years

	No sanction (n=14,913)		Community sanction (n=72)		Diversion (n=25)		Current imprisonment (n<5)		Prior imprisonment (n=14)		Total (n=15,027)	
	**n**	**(%)**	**n**	**(%)**	**n**	**(%)**	**n**	**(%)**	**n**	**(%)**	**n**	**(%)**
**Sex**												
Female or other[a]	9,035	(60.6)	11	(15.3)	5	(20.0)	0	(0.0)	0	(0.0)	9,051	(60.2)
**Age group**												
≥65 years	14,913	(100.0)	72	(100.0)	25	(100.0)	<5	NS	14	(100.0)	15,027	(100.0)
**Aboriginal and/or Torres Strait Islander identity**												
Yes	313	(2.1)	5	(6.9)	<5	NS	0	(0.0)	6	(42.9)	326	(2.2)
No	14,573	(97.7)	67	(93.1)	23	(92.0)	<5	NS	8	(57.1)	14,674	(97.7)
Missing or unknown	27	(0.2)	0	(0.0)	0	(0.0)	0	(0.0)	0	(0.0)	27	(0.2)
**Residential Index of Relative Socioeconomic Disadvantage**												
Least disadvantaged	6,343	(42.5)	26	(36.1)	12	(48.0)	<5	NS	<5	NS	6,385	(42.5)
Moderately disadvantaged	4,594	(30.8)	23	(31.9)	7	(28.0)	0	(0.0)	7	(50.0)	4,631	(30.8)
Most disadvantaged	3,566	(23.9)	18	(25.0)	6	(24.0)	<5	NS	5	(35.7)	3,596	(23.9)
Interstate resident	225	(1.5)	<5	NS	0	(0.0)	0	(0.0)	0	(0.0)	226	(1.5)
Missing or unknown	185	(1.2)	<5	NS	0	(0.0)	0	(0.0)	0	(0.0)	189	(1.3)

© 2024 Spike E et al. *JAMA Network Open*

	No sanction (n=14,913)		Community sanction (n=72)		Diversion (n=25)		Current imprisonment (n<5)		Prior imprisonment (n=14)		Total (n=15,027)	
**Residential remoteness**												
Major cities	10,812	(72.5)	55	(76.4)	20	(80.0)	<5	NS	8	(57.1)	10,898	(72.5)
Inner regional	2,865	(19.2)	11	(15.3)	<5	NS	0	(0.0)	<5	NS	2,882	(19.2)
Outer regional/remote	826	(5.5)	<5	NS	<5	NS	0	(0.0)	<5	NS	832	(5.5)
Interstate resident	225	(1.5)	<5	NS	0	(0.0)	0	(0.0)	0	(0.0)	226	(1.5)
Missing or unknown	185	(1.2)	<5	NS	0	(0.0)	0	(0.0)	0	(0.0)	189	(1.3)
**Married (including de facto)**												
Yes	5,112	(34.3)	14	(19.4)	<5	NS	<5	NS	5	(35.7)	5,136	(34.2)
No	8,614	(57.8)	50	(69.4)	18	(72.0)	<5	NS	7	(50.0)	8,691	(57.8)
Missing or unknown	1,187	(8.0)	8	(11.1)	<5	NS	0	(0.0)	<5	NS	1,200	(8.0)
**Problematic drug use**												
Yes	903	(6.1)	26	(36.1)	7	(28.0)	<5	NS	6	(42.9)	945	(6.3)
**Problematic alcohol use**												
Yes	1,412	(9.5)	18	(25.0)	7	(28.0)	<5	NS	5	(35.7)	1,443	(9.6)
**Involuntary index admission**												
Yes	5,661	(38.0)	35	(48.6)	13	(52.0)	<5	NS	7	(50.0)	5,718	(38.1)
**Charlson Comorbidity Index (past year of age)**												

© 2024 Spike E et al. *JAMA Network Open*

	No sanction (n=14,913)		Community sanction (n=72)		Diversion (n=25)		Current imprisonment (n<5)		Prior imprisonment (n=14)		Total (n=15,027)	
0	11,549	(77.4)	57	(79.2)	16	(64.0)	0	(0.0)	9	(64.3)	11,631	(77.4)
1	1,579	(10.6)	<5	NS	<5	NS	0	(0.0)	<5	NS	1,587	(10.6)
≥2	1,785	(12.0)	12	(16.7)	6	(24.0)	<5	NS	<5	NS	1,809	(12.0)
**Offense conviction history**												
None	14,283	(95.8)	42	(58.3)	14	(56.0)	0	(0.0)	5	(35.7)	14,344	(95.5)
Non-violent offenses only	294	(2.0)	8	(11.1)	<5	NS	<5	NS	<5	NS	309	(2.1)
Violent offenses	336	(2.3)	22	(30.6)	8	(32.0)	<5	NS	7	(50.0)	374	(2.5)
Unknown offenses only	0	(0.0)	0	(0.0)	0	(0.0)	0	(0.0)	0	(0.0)	0	(0.0)
**Died**												
Yes	5,540	(37.1)	11	(15.3)	11	(44.0)	<5	NS	<5	NS	5,565	(37.0)

NS=not shown due to corresponding n<5.

[a]Other category not reported separately due to having less than 5 participants in the entire cohort; other included intersex.

eFigure 1. Age- and Sex-Specific All-Cause Mortality Rates Among People With Psychosis Aged 18 to 64 Years by Recent (Past 2 Years) Criminal Sanction Type (n=74,841)†

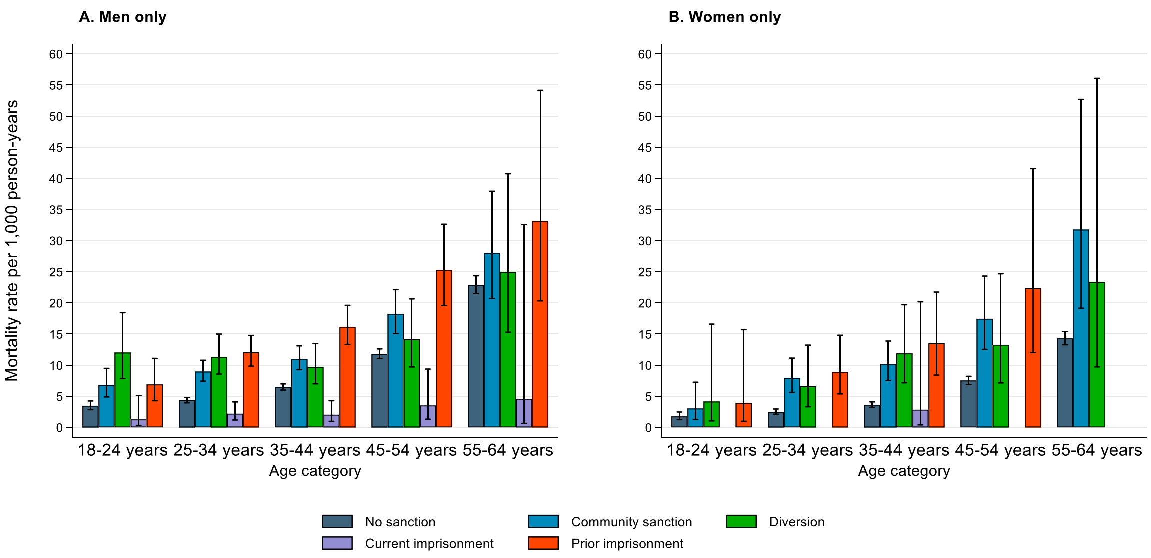

†For graphical purposes, two deaths were excluded from the 'prior imprisonment' category in women aged 55-64 years. Error bars represent 95% confidence intervals.

© 2024 Spike E et al. *JAMA Network Open*

eTable 5. Age- and Sex-Specific All-Cause Mortality Rates by Recent (Past 2 Years) Criminal Sanction Type

	No sanction			Community sanction			Diversion			Current imprisonment			Prior imprisonment		
	**D**	**PY**	**MR (95% CI)**	**D**	**PY**	**MR (95% CI)**	**D**	**PY**	**MR (95% CI)**	**D**	**PY**	**MR (95% CI)**	**D**	**PY**	**MR (95% CI)**
**Men**															
18-24 years	96	27,619	3.48 (2.85-4.25)	35	5,138	6.81 (4.89-9.49)	21	1,748	12.02 (7.83-18.43)	<5	NS	1.28 (0.32-5.11)	17	2,465	6.90 (4.29-11.09)
25-34 years	396	91,049	4.35 (3.94-4.80)	111	12,391	8.96 (7.44-10.79)	49	4,324	11.33 (8.57-14.99)	10	4,540	2.20 (1.19-4.09)	93	7,713	12.06 (9.84-14.77)
35-44 years	664	102,623	6.47 (6.00-6.98)	129	11,709	11.02 (9.27-13.09)	36	3,709	9.71 (7.00-13.46)	7	3,426	2.04 (0.97-4.29)	103	6,371	16.17 (13.33-19.61)
45-54 years	905	76,622	11.81 (11.07-12.61)	104	5,698	18.25 (15.06-22.12)	27	1,907	14.16 (9.71-20.64)	<5	NS	3.52 (1.32-9.37)	59	2,333	25.29 (19.59-32.64)
55-64 years	982	42,922	22.88 (21.49-24.36)	42	1,498	28.03 (20.72-37.93)	16	641	24.96 (15.29-40.75)	<5	NS	4.59 (0.65-32.60)	16	482	33.17 (20.32-54.15)
≥65 years	2199	30,733	71.55 (68.62-74.61)	8	317	25.26 (12.63-50.51)	9	148	60.93 (31.70-117.11)	<5	NS	43.22 (6.09-306.84)	<5	NS	35.89 (8.98-143.49)
**Women**															
18-24 years	33	18,802	1.76 (1.25-2.47)	5	1,654	3.02 (1.26-7.26)	<5	NS	4.15 (1.04-16.59)	0	188	..	<5	NS	3.92 (0.98-15.69)
25-34 years	142	56,746	2.50 (2.12-2.95)	33	4,169	7.92 (5.63-11.13)	8	1,210	6.61 (3.31-13.22)	0	591	..	15	1,680	8.93 (5.38-14.81)
35-44 years	266	73,250	3.63 (3.22-4.10)	41	4,013	10.22 (7.52-13.87)	15	1,263	11.88 (7.16-19.70)	<5	NS	2.84 (0.40-20.18)	17	1,258	13.51 (8.40-21.74)
45-54 years	507	67,394	7.52 (6.90-8.21)	35	2,005	17.45 (12.53-24.31)	10	753	13.28 (7.14-24.68)	0	117	..	10	447	22.36 (12.03-41.56)

© 2024 Spike E et al. *JAMA Network Open*

	No sanction			Community sanction			Diversion			Current imprisonment			Prior imprisonment		
	**D**	**PY**	**MR (95% CI)**	**D**	**PY**	**MR (95% CI)**	**D**	**PY**	**MR (95% CI)**	**D**	**PY**	**MR (95% CI)**	**D**	**PY**	**MR (95% CI)**
55-64 years	701	49,047	14.29 (13.27-15.39)	15	472	31.77 (19.15-52.69)	5	214	23.34 (9.72-56.08)	0	25	..	<5	NS	33.24 (8.31-132.90)
≥65 years	3341	53,446	62.51 (60.43-64.67)	<5	NS	34.42 (11.10-106.73)	<5	NS	60.06 (15.02-240.13)	0	1	..	0	6	..
**Both**															
18-24 years	129	46,421	2.78 (2.34-3.30)	40	6,792	5.89 (4.32-8.03)	23	2,230	10.32 (6.86-15.52)	<5	NS	1.14 (0.29-4.57)	19	2,975	6.39 (4.07-10.01)
25-34 years	538	147,795	3.64 (3.35-3.96)	144	16,560	8.70 (7.39-10.24)	57	5,534	10.30 (7.95-13.35)	10	5,131	1.95 (1.05-3.62)	108	9,393	11.50 (9.52-13.88)
35-44 years	930	175,872	5.29 (4.96-5.64)	170	15,723	10.81 (9.30-12.57)	51	4,971	10.26 (7.80-13.50)	8	3,778	2.12 (1.06-4.23)	120	7,629	15.73 (13.15-18.81)
45-54 years	1412	144,016	9.80 (9.31-10.33)	139	7,703	18.05 (15.28-21.31)	37	2,660	13.91 (10.08-19.20)	<5	NS	3.19 (1.20-8.50)	69	2,780	24.82 (19.60-31.42)
55-64 years	1683	91,969	18.30 (17.45-19.20)	57	1,971	28.93 (22.31-37.50)	21	855	24.56 (16.01-37.66)	<5	NS	4.12 (0.58-29.24)	18	542	33.18 (20.91-52.66)
≥65 years	5540	84,179	65.81 (64.10-67.57)	11	404	27.24 (15.08-49.19)	11	181	60.77 (33.66-109.74)	<5	NS	41.90 (5.90-297.45)	<5	NS	32.26 (8.07-129.01)

D=deaths, PY=person years, MR=mortality rate, CI=confidence interval, NS=not shown due to corresponding n<5.

© 2024 Spike E et al. *JAMA Network Open*

eFigure 2. Age- and Sex-Specific External-Cause Mortality Rates Among People With Psychosis Aged 18 to 64 Years by Recent (Past 2 Years) Criminal Sanction Type (n=74,841)†

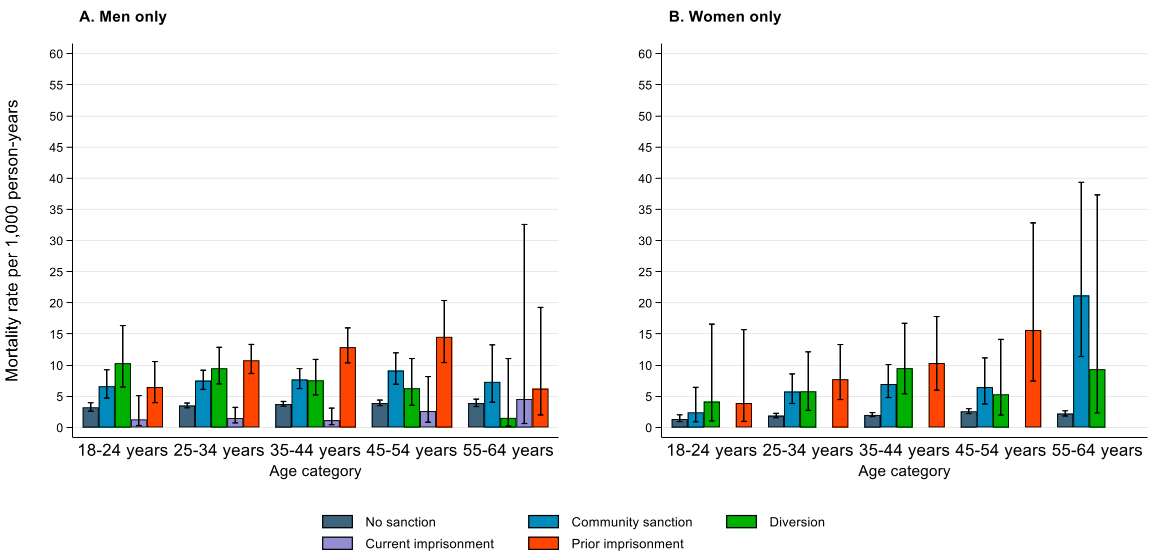

†For graphical purposes, one death was excluded from the 'prior imprisonment' category in women aged 55-64 years. Error bars represent 95% confidence intervals.

© 2024 Spike E et al. *JAMA Network Open*

eTable 6. Age- and Sex-Specific External-Cause Mortality Rates by Recent (Past 2 Years) Criminal Sanction Type

	No sanction			Community sanction			Diversion			Current imprisonment			Prior imprisonment		
	**D**	**PY**	**MR (95% CI)**	**D**	**PY**	**MR (95% CI)**	**D**	**PY**	**MR (95% CI)**	**D**	**PY**	**MR (95% CI)**	**D**	**PY**	**MR (95% CI)**
**Men**															
18-24 years	89	27,619	3.22 (2.62-3.97)	34	5,138	6.62 (4.73-9.26)	18	1,748	10.30 (6.49-16.35)	<5	NS	1.28 (0.32-5.11)	16	2,465	6.49 (3.98-10.59)
25-34 years	320	91,049	3.51 (3.15-3.92)	93	12,391	7.51 (6.13-9.20)	41	4,324	9.48 (6.98-12.88)	7	4,540	1.54 (0.74-3.23)	83	7,713	10.76 (8.68-13.34)
35-44 years	389	102,623	3.79 (3.43-4.19)	90	11,709	7.69 (6.25-9.45)	28	3,709	7.55 (5.21-10.93)	<5	NS	1.17 (0.44-3.11)	82	6,371	12.87 (10.37-15.98)
45-54 years	301	76,622	3.93 (3.51-4.40)	52	5,698	9.13 (6.95-11.98)	12	1,907	6.29 (3.57-11.08)	<5	NS	2.64 (0.85-8.18)	34	2,333	14.57 (10.41-20.39)
55-64 years	168	42,922	3.91 (3.36-4.55)	11	1,498	7.34 (4.07-13.26)	<5	NS	1.56 (0.22-11.08)	<5	NS	4.59 (0.65-32.60)	<5	NS	6.22 (2.01-19.29)
≥65 years	135	30,733	4.39 (3.71-5.20)	0	317	..	<5	NS	20.31 (6.55-62.98)	0	23	..	0	56	..
**Women**															
18-24 years	26	18,802	1.38 (0.94-2.03)	<5	NS	2.42 (0.91-6.44)	<5	NS	4.15 (1.04-16.59)	0	188	..	<5	NS	3.92 (0.98-15.69)
25-34 years	107	56,746	1.89 (1.56-2.28)	24	4,169	5.76 (3.86-8.59)	7	1,210	5.79 (2.76-12.14)	0	591	..	13	1,680	7.74 (4.49-13.32)
35-44 years	149	73,250	2.03 (1.73-2.39)	28	4,013	6.98 (4.82-10.10)	12	1,263	9.50 (5.40-16.73)	0	352	..	13	1,258	10.33 (6.00-17.80)
45-54 years	175	67,394	2.60 (2.24-3.01)	13	2,005	6.48 (3.76-11.16)	<5	NS	5.31 (1.99-14.15)	0	117	..	7	447	15.65 (7.46-32.83)

© 2024 Spike E et al. *JAMA Network Open*

	No sanction			Community sanction			Diversion			Current imprisonment			Prior imprisonment		
	**D**	**PY**	**MR (95% CI)**	**D**	**PY**	**MR (95% CI)**	**D**	**PY**	**MR (95% CI)**	**D**	**PY**	**MR (95% CI)**	**D**	**PY**	**MR (95% CI)**
55-64 years	109	49,047	2.22 (1.84-2.68)	10	472	21.18 (11.39-39.36)	<5	NS	9.34 (2.33-37.33)	0	25	..	<5	NS	16.62 (2.34-117.98)
≥65 years	139	53,446	2.60 (2.20-3.07)	0	87	..	<5	NS	30.03 (4.23-213.17)	0	1	..	0	6	..
**Both**															
18-24 years	115	46,421	2.48 (2.06-2.97)	38	6,792	5.59 (4.07-7.69)	20	2,230	8.97 (5.79-13.90)	<5	NS	1.14 (0.29-4.57)	18	2,975	6.05 (3.81-9.60)
25-34 years	427	147,795	2.89 (2.63-3.18)	117	16,560	7.07 (5.89-8.47)	48	5,534	8.67 (6.54-11.51)	7	5,131	1.36 (0.65-2.86)	96	9,393	10.22 (8.37-12.48)
35-44 years	538	175,872	3.06 (2.81-3.33)	118	15,723	7.51 (6.27-8.99)	40	4,971	8.05 (5.90-10.97)	<5	NS	1.06 (0.40-2.82)	95	7,629	12.45 (10.18-15.23)
45-54 years	476	144,016	3.31 (3.02-3.62)	65	7,703	8.44 (6.62-10.76)	16	2,660	6.01 (3.68-9.82)	<5	NS	2.39 (0.77-7.42)	41	2,780	14.75 (10.86-20.03)
55-64 years	277	91,969	3.01 (2.68-3.39)	21	1,971	10.66 (6.95-16.35)	<5	NS	3.51 (1.13-10.88)	<5	NS	4.12 (0.58-29.24)	<5	NS	7.37 (2.77-19.65)
≥65 years	274	84,179	3.25 (2.89-3.66)	0	404	..	<5	NS	22.10 (8.29-58.88)	0	24	..	0	62	..

D=deaths, PY=person years, MR=mortality rate, CI=confidence interval, NS=not shown due to corresponding n<5.

© 2024 Spike E et al. *JAMA Network Open*

eTable 7. All-Cause Mortality Hazard Ratios by Recent (Past 2 Years) Criminal Sanction Type Among Men With Psychosis Aged 18 to 64 Years (n=44,287)

	**Univariable**			**Model 1**			**Model 2**			**Model 3**		
**Criminal sanction type**	**HR**	**95% CI**	**P value**	**HR**	**95% CI**	**P value**	**HR**	**95% CI**	**P value**	**HR**	**95% CI**	**P value**
**No sanction**	1.00	(ref)	<0.0001	1.00	(ref)	<0.0001	1.00	(ref)	<0.0001	1.00	(ref)	<0.0001
**Community sanction**	1.28	(1.15-1.42)		1.61	(1.45-1.78)		1.58	(1.42-1.75)		1.30	(1.16-1.45)	
**Diversion**	1.35	(1.14-1.59)		1.65	(1.39-1.94)		1.62	(1.37-1.91)		1.41	(1.19-1.66)	
**Current imprisonment**	0.25	(0.17-0.37)		0.35	(0.23-0.52)		0.35	(0.23-0.53)		0.27	(0.18-0.41)	
**Prior imprisonment**	1.66	(1.47-1.87)		2.26	(1.99-2.55)		2.24	(1.98-2.54)		1.71	(1.49-1.95)	

Model 1 adjusted for age. Model 2 adjusted for Model 1 covariates plus Aboriginal and/or Torres Strait Islander identity, marital status, residential Index of Relative Socioeconomic Disadvantage, residential remoteness. Model 3 adjusted for Model 2 covariates plus history of problematic drug use, history of problematic alcohol use, involuntary index admission, Charlson Comorbidity Index score, offense history. HR=hazard ratio, CI=confidence interval.

© 2024 Spike E et al. *JAMA Network Open*

eTable 8. All-Cause Mortality Hazard Ratios by Recent (Past 2 Years) Criminal Sanction Type Among Women With Psychosis Aged 18 to 64 Years (n=30,554)

	**Univariable**			**Model 1**			**Model 2**			**Model 3**		
**Criminal sanction type**	**HR**	**95% CI**	**P value**	**HR**	**95% CI**	**P value**	**HR**	**95% CI**	**P value**	**HR**	**95% CI**	**P value**
**No sanction**	1.00	(ref)	<0.0001	1.00	(ref)	<0.0001	1.00	(ref)	<0.0001	1.00	(ref)	<0.0001
**Community sanction**	1.68	(1.41-2.01)		2.53	(2.11-3.04)		2.34	(1.94-2.82)		1.62	(1.33-1.97)	
**Diversion**	1.65	(1.20-2.26)		2.26	(1.65-3.09)		2.09	(1.53-2.87)		1.54	(1.12-2.13)	
**Current imprisonment**	0.13	(0.02-0.90)		0.23	(0.03-1.62)		0.20	(0.03-1.45)		0.11	(0.02-0.80)	
**Prior imprisonment**	1.89	(1.41-2.53)		3.26	(2.42-4.38)		2.96	(2.19-4.00)		1.68	(1.23-2.31)	

Model 1 adjusted for age. Model 2 adjusted for Model 1 covariates plus Aboriginal and/or Torres Strait Islander identity, marital status, residential Index of Relative Socioeconomic Disadvantage, residential remoteness. Model 3 adjusted for Model 2 covariates plus history of problematic drug use, history of problematic alcohol use, involuntary index admission, Charlson Comorbidity Index score, offense history. HR=hazard ratio, CI=confidence interval.

© 2024 Spike E et al. *JAMA Network Open*

eTable 9. External-Cause Mortality Hazard Ratios **by** Recent (Past 2 Years) Criminal Sanction Type Among Men With Psychosis Aged 18 to 64 Years (n=44,287)

	**Univariable**			**Model 1**			**Model 2**			**Model 3**		
**Criminal sanction type**	**HR**	**95% CI**	**P value**	**HR**	**95% CI**	**P value**	**HR**	**95% CI**	**P value**	**HR**	**95% CI**	**P value**
**No sanction**	1.00	(ref)	<0.0001	1.00	(ref)	<0.0001	1.00	(ref)	<0.0001	1.00	(ref)	<0.0001
**Community sanction**	1.93	(1.69-2.20)		1.99	(1.74-2.26)		2.01	(1.77-2.30)		1.71	(1.49-1.97)	
**Diversion**	2.06	(1.68-2.53)		2.12	(1.73-2.60)		2.13	(1.74-2.62)		1.97	(1.59-2.43)	
**Current imprisonment**	0.40	(0.25-0.65)		0.42	(0.26-0.68)		0.45	(0.28-0.73)		0.38	(0.23-0.61)	
**Prior imprisonment**	2.88	(2.50-3.33)		2.98	(2.58-3.45)		3.13	(2.70-3.63)		2.57	(2.18-3.02)	

Model 1 adjusted for age. Model 2 adjusted for Model 1 covariates plus Aboriginal and/or Torres Strait Islander identity, marital status, residential Index of Relative Socioeconomic Disadvantage, residential remoteness. Model 3 adjusted for Model 2 covariates plus history of problematic drug use, history of problematic alcohol use, involuntary index admission, Charlson Comorbidity Index score, offense history. HR=hazard ratio, CI=confidence interval.

© 2024 Spike E et al. *JAMA Network Open*

eTable 10. External-Cause Mortality Hazard Ratios by Recent (Past 2 Years) Criminal Sanction Type Among Women With Psychosis Aged 18 to 64 Years (n=30,554)

	Univariable			Model 1			Model 2			Model 3		
**Criminal sanction type**	**HR**	**95% CI**	**P value**	**HR**	**95% CI**	**P value**	**HR**	**95% CI**	**P value**	**HR**	**95% CI**	**P value**
**No sanction**	1.00	(ref)	<0.0001	1.00	(ref)	<0.0001	1.00	(ref)	<0.0001	1.00	(ref)	<0.0001
**Community sanction**	2.89	(2.28-3.66)		3.17	(2.50-4.03)		3.16	(2.47-4.03)		2.19	(1.68-2.87)	
**Diversion**	3.14	(2.14-4.63)		3.35	(2.27-4.94)		3.22	(2.18-4.75)		2.52	(1.68-3.77)	
**Current imprisonment**†	NR	NR		NR	NR		NR	NR		NR	NR	
**Prior imprisonment**	4.19	(2.99-5.86)		4.74	(3.37-6.67)		4.82	(3.39-6.84)		3.05	(2.09-4.46)	

Model 1 adjusted for age. Model 2 adjusted for Model 1 covariates plus Aboriginal and/or Torres Strait Islander identity, marital status, residential Index of Relative Socioeconomic Disadvantage, residential remoteness. Model 3 adjusted for Model 2 covariates plus history of problematic drug use, history of problematic alcohol use, involuntary index admission, Charlson Comorbidity Index score, offense history. HR=hazard ratio, CI=confidence interval, NR=not reported. †Estimates for the 'Current imprisonment' category omitted due to zero events in this category.

© 2024 Spike E et al. *JAMA Network Open*

eReferences.

1. *Mental Health (Forensic Provisions) Act 1990 No 10* (NSW) pt 3. Accessed August 18, 2024. Available from: https://legislation.nsw.gov.au/view/html/repealed/current/act-1990-010.

2. *Mental Health and Cognitive Impairment Forensic Provisions Act 2020 No 12* (NSW) pt 2 div 2 sec 14. Available from: https://www.legislation.nsw.gov.au/view/html/inforce/current/act-2020-012#sec.14

3. Kariminia A., Butler T.G., Corben S.P., Levy M.H., Grant L., Kaldor J.M., et al. Extreme cause-specific mortality in a cohort of adult prisoners - 1988 to 2002: a data-linkage study. *Int J Epidemiol*. 2007;36(2):310–6. doi:10.1093/ije/dyl225

4. Spaulding AC, Seals RM, McCallum VA, Perez SD, Brzozowski AK, Steenland NK. Prisoner Survival Inside and Outside of the Institution: Implications for Health-Care Planning. *Am J Epidemiol*. 2011;173(5):479–87. doi:10.1093/aje/kwq422

5. Kinner S.A., Forsyth S., Williams G. Systematic review of record linkage studies of mortality in ex-prisoners: why (good) methods matter. *Addiction*. 2013;108(1):38–49. doi:10.1111/add.12010

6. Australian Bureau of Statistics. *2033.0.55.001 - Socio-economic Indexes for Areas (SEIFA), Data Cube only, 2011*. Australian Bureau of Statistics; March 28, 2013. Accessed July 24, 2023. https://www.abs.gov.au/AUSSTATS/subscriber.nsf/log?openagent&2033.0.55.001%20sa1%20indexes.xls&2033.0.55.001&Data%20Cubes&9828E2819C30D96DCA257B43000E923E&0&2011&05.04.2013&Latest.

7. Quan H, Li B, Couris CM, et al. Updating and Validating the Charlson Comorbidity Index and Score for Risk Adjustment in Hospital Discharge Abstracts Using Data From 6 Countries. *Am J Epidemiol*. 2011;173(6):676–682. doi:10.1093/aje/kwq433

8. NSW Bureau of Crime Statistics and Research. *NSW Criminal Justice Aboriginal Over-representation. Quarterly Report. March 2023 - Summary*. NSW Bureau of Crime Statistics and Research; June 2023. Accessed August 18, 2024. https://www.bocsar.nsw.gov.au/Documents/Aboriginal%20over-rep/AOR-Summary-March-2023.pdf

9. Australian Government, Australian Institute of Health and Welfare, and National Indigenous Australians Agency. Aboriginal and Torres Strait Islander Health Performance Framework. *Tier 1 - Health status and outcomes. 1.22 All-cause age-standardised death rates*. Australian Institute of Health and Welfare; 2024. Accessed August 18, 2024. https://www.indigenoushpf.gov.au/measures/1-22-all-cause-age-standardised-death-rates#:~:text=In%202015%E2%80%932019%2C%20Indigenous%20Australians,deaths%20of%20non%E2%80%91Indigenous%20Australians.

10. Nelson M, Lim K, Boyd J, et al. Accuracy of reporting of Aboriginality on administrative health data collections using linked data in NSW, Australia. *BMC Med Res Methodol*. 2020;20:1–8. doi:10.1186/s12874-020-01152-2

11. Talbot D, Massamba VK. A descriptive review of variable selection methods in four epidemiologic journals: there is still room for improvement. *Eur J Epidemiol.* 2019;34(8):725–30. doi:10.1007/s10654-019-00529-y
