# Supplementary material for: Mortality, Criminal Sanctions, and Court Diversion in People With Psychosis
Source: JAMA Netw Open. 2024 Oct 31;7(10):e2442146. doi: 10.1001/jamanetworkopen.2024.42146 (PMC11528309; doi:10.1001/jamanetworkopen.2024.42146)
Supplement: Supplement 2. — Data Sharing Statement [file jamanetwopen-e2442146-s002.pdf]

Data Sharing Statement

Spike. Mortality, Criminal Sanctions, and Court Diversion in People With Psychosis. *JAMA Netw Open*. Published October 31, 2024. doi:10.1001/jamanetworkopen.2024.42146

Data
Data available: No

Additional Information
Explanation for why data not available: The data used in this study will not be made publicly available as this is a condition of the ethical approvals received for this study. With the exception of OIMS, data dictionaries for the underlying data collections used in the study are available from the NSW Centre for Health Record Linkage website (https://www.cherel.org.au/datasets) or the NSW Bureau of Crime Statistics and Research website (https://www.bocsar.nsw.gov.au/Documents/guidelines_and_form_to_access_unitrecord_reoffending_data.doc). Statistical code will be available from the corresponding author Dr Erin Spike (espike@kirby.unsw.edu.au) upon request.
